# The C-terminal tail of the yeast mitochondrial transcription factor Mtf1 coordinates template strand alignment, DNA scrunching and timely transition into elongation

**DOI:** 10.1093/nar/gkaa040

**Published:** 2020-01-25

**Authors:** Urmimala Basu, Seung-Won Lee, Aishwarya Deshpande, Jiayu Shen, Byeong-Kwon Sohn, Hayoon Cho, Hajin Kim, Smita S Patel

**Affiliations:** 1 Department of Biochemistry and Molecular Biology, Rutgers University, Robert Wood Johnson Medical School, Piscataway, NJ 08854, USA; 2 Graduate School of Biomedical Sciences at Robert Wood Johnson Medical School of the Rutgers University, USA; 3 School of Life Sciences, Ulsan National Institute of Science and Technology, Ulsan, Republic of Korea; 4 Center for Genomic Integrity, Institute for Basic Science, Ulsan, Republic of Korea

## Abstract

Mitochondrial RNA polymerases depend on initiation factors, such as TFB2M in humans and Mtf1 in yeast *Saccharomyces cerevisiae*, for promoter-specific transcription. These factors drive the melting of promoter DNA, but how they support RNA priming and growth was not understood. We show that the flexible C-terminal tails of Mtf1 and TFB2M play a crucial role in RNA priming by aiding template strand alignment in the active site for high-affinity binding of the initiating nucleotides. Using single-molecule fluorescence approaches, we show that the Mtf1 C-tail promotes RNA growth during initiation by stabilizing the scrunched DNA conformation. Additionally, due to its location in the path of the nascent RNA, the C-tail of Mtf1 serves as a sensor of the RNA–DNA hybrid length. Initially, steric clashes of the Mtf1 C-tail with short RNA–DNA hybrids cause abortive synthesis but clashes with longer RNA-DNA trigger conformational changes for the timely release of the promoter DNA to commence the transition into elongation. The remarkable similarities in the functions of the C-tail and σ3.2 finger of the bacterial factor suggest mechanistic convergence of a flexible element in the transcription initiation factor that engages the DNA template for RNA priming and growth and disengages when needed to generate the elongation complex.

## INTRODUCTION

The mitochondrial genomes of eukaryotes are transcribed by RNA polymerases (RNAPs) that are distinct from nuclear RNAPs and homologous to single-subunit bacteriophage T7/T3 RNAP (1). The mitochondrial RNAPs of *Saccharomyces cerevisiae* (yeast) and human are the best-studied examples in this class. Unlike T7 RNAP, the mitochondrial RNAPs depend on transcription factors for promoter-specific transcription, a known feature of multisubunit RNAPs. The yeast mitochondrial RNAP, Rpo41, requires Mitochondrial Transcription Factor 1 (Mtf1) to initiate promoter-specific transcription (2,3) and the human POLRMT requires Transcription Factor B2 mitochondrial (TFB2M) and Transcription Factor A mitochondrial (TFAM) for transcription initiation (4–7).

**Figure 1.**
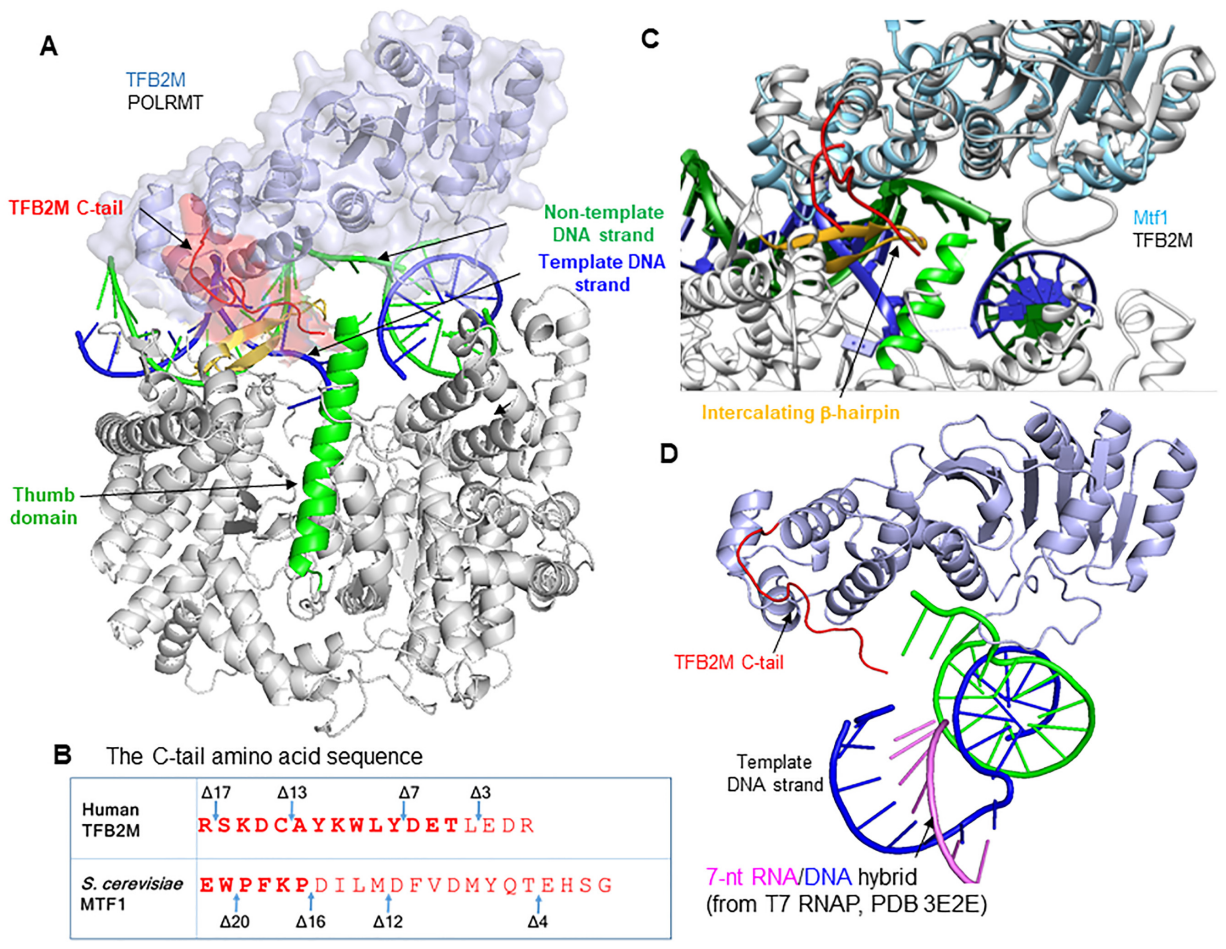
Location of the C-terminal tail of the initiation factors (TFB2M and Mtf1) in the open complex. (**A**) Structure of the human mitochondrial RNAP open complex (PDB: 6ERP) shows POLRMT (in grey), TFB2M (in light blue), template DNA strand (in navy blue), non-template DNA strand (in green) and the C-tail of TFB2M (in red) interacting with the POLRMT intercalating β-helix (in yellow) and thumb domain helix (in green). TFAM is not shown. The promoter DNA is severely bent around the initiation site. (**B**) The amino acid sequence of the C-tail of TFB2M and Mtf1. The missing amino acids in the crystal structures are shown in light letters. Mtf1 is missing 16 aa of the C-tail, and TFB2M is missing 4 aa of the C-tail. (**C**) Yeast Mtf1 (PDB: 1I4W) is aligned with human TFB2M bound to POLRMT in the open complex. The C-tails are shown in red. (**D**) Stalled +7 initiation complex of T7 RNAP (PDB: 3E2E) was aligned with POLRMT (PDB: 6ERP) and the position of the 7 base-pair RNA–DNA hybrid (pink-blue) is shown to illustrate a potential clash of the RNA–DNA hybrid with the C-tail of TFB2M (the closest distance between the 4 aa deleted TFB2M C-tail to the 5′-end of the 7 bp RNA–DNA is ∼8 Ä) (13,16).

Mtf1 and TFB2M are evolutionarily related to RNA methyltransferases, but as transcription initiation factors, they facilitate promoter-specific transcription initiation (3,6–9). Both initiation factors drive the melting of the promoter DNA by binding to the non-template strand and trapping the melted strand in their nucleic acid binding pocket (10–13). Recent structural studies show that the N-terminal domain of human TFB2M binds to the non-template strand while the C-terminal domain provides upstream promoter contacts with the –7 promoter region (Figure 1A) (13). Curiously, TFB2M and Mtf1 proteins contain a flexible C-terminal tail region (Figure 1B), which is ordered in the open complex structure of POLRMT (Figure 1C). The C-tail of TFB2M contacts the intercalating β-hairpin and the thumb domain of POLRMT and is positioned in the path of the nascent RNA (Supplementary Figure S1). Crosslinking and DNA cleavage studies have shown a similar location of the C-tail of Mtf1 positioned between the melted DNA strands and in the proximity of the −3/−4 template bases in the open complex (14,15).

The location of the C-tail near the active site suggests that it has a role in transcription initiation. Being in the path of the growing RNA also indicates that at some point during initial transcription, the C-tail will sterically clash with the RNA–DNA hybrid. A structural alignment of POLRMT (PDB: 6ERP) with T7 RNAP bound to a 7-bp RNA–DNA hybrid (PDB: 2E3E) (16) shows that the C-tail will clash with the 7-bp RNA–DNA hybrid (Figure 1D). Such an arrangement is reminiscent of the σ3.2 finger and *B*-reader of the initiation factors of bacterial and nuclear RNAPs, respectively, which are present in a similar location in the path of the initially growing RNA transcript (17,18). The σ3.2 finger is also essential for efficient RNA priming during transcription initiation (18–21). The bacterial and mitochondrial initiation factors are evolutionarily unrelated and there is no structural homology between the two factors. However, σ3.2 finger and C-tail have similar locations near the active site of the core RNAP in the initiation complex, which brings up an intriguing possibility that the two elements might be functionally homologous.

Herein, we have used ensemble and single-molecule fluorescence-based methods to investigate the role of the C-terminal tail region of Mtf1 and TFB2M. Contrary to previous studies (15), we show that Mtf1 C-tail is not required for promoter melting. Instead, our studies show that the C-tail regions of Mtf1 and TFB2M are critical for RNA priming and growth. Our recent studies showed that the yeast mitochondrial RNAP catalyzes transcription initiation by the DNA scrunching mechanism (22). Herein, we show that the C-tail region is needed to stabilize the scrunched DNA throughout transcription initiation. Consistent with the location of the Mtf1 C-tail in the path of the growing RNA, we show that Mtf1 C-tail deletion reduces abortive synthesis, but interestingly, it also alters the timing of transition into elongation. Based on our studies, we propose that the C-tail region sterically clashes with the RNA–DNA hybrid to produce abortive RNAs, but later on, the clashes with the more stable RNA–DNA trigger the timely release of upstream promoter contacts and transition into elongation. With these studies, we present the first detailed model of transcription initiation by the mitochondrial RNAP with the C-tail region as a key player in coordinating template strand alignment, DNA scrunching, and promoter release events for efficient transcription initiation and timely transition into elongation. This is a case of mechanistic convergence where evolutionarily unrelated C-tail regions of the mitochondrial initiation factors and σ3.2 finger of the bacterial initiation factor are preforming very similar functions in promoter-specific transcription initiation.

## MATERIALS AND METHODS

### Purification of proteins

The expression and purification of Rpo41, Mtf1, and C-tail deletion mutants were carried out as reported previously (23–25). The expression and purification of POLRMT, TFAM, TFB2M and C-tail deletion mutants was carried out as reported previously (6). The proteins were stored in 10% or 50% glycerol at −80°C. The molar concentrations of the proteins were determined from absorbance measurements at 280 nm using guanidium–HCl buffer and molar extinction coefficients.

### Preparation of DNA templates

DNA oligodeoxynucleotides were custom synthesized with biotin and amino modifications and purified by HPLC (Integrated DNA Technologies, Coralville, IA, USA). DNAs were labeled with Cy3 or Cy5 NHS ester fluorophores at the amine groups by standard methods (Lumiprobe, USA) and unreacted dyes were removed by ethanol precipitation. Pairs of single-stranded DNAs were mixed in 1:1 ratio, annealed at 95°C for 1 min, and cooled over an hour to room temperature to make the duplex DNA molecules.

### Fluorescence anisotropy titrations to measure the binding of Rpo41-Mtf1 to promoter DNA

Fluorescence anisotropy measurements were carried out on Fluoro-Max-4 spectrofluorometer (Jobin Yvon-Spex Instruments S.A., Inc.) at 25°C. TAMRA fluorophore-labeled promoter DNA (5 nM with TAMRA label on the 5′ end of template strand) was titrated with 1:1.2 ratio of Rpo41 and Mtf1 in reaction buffer A (50 mM Tris-acetate pH 7.5, 100 mM potassium glutamate, 10 mM magnesium acetate, 0.01% Tween-20). Anisotropy values (*r*_obs_) were recorded with excitation at 555 nm and emission at 580 nm. The *r*_obs_ versus protein concentration plots were fit to Equation (1) to obtain the equilibrium dissociation constant (*K*_d_).

(1) $$\begin{eqnarray*}{r_{{\rm{obs}}}} = \frac{{\left( {{K_{\rm{d}}} + \left[ {{P_{\rm{t}}}} \right] + \left[ {{D_{\rm{t}}}} \right]} \right) - \sqrt {{{\left( {{K_{\rm{d}}} + \left[ {{P_{\rm{t}}}} \right] + \left[ {{D_{\rm{t}}}} \right]} \right)}^2} - 4 \cdot {P_{\rm{t}}} \cdot {D_{\rm{b}}}} }}{{2\left[ {{D_{\rm{t}}}} \right]}} \cdot \left( {{r_{\rm{b}}} - {r_{\rm{f}}}} \right) + {r_{\rm{f}}}\nonumber\\ \end{eqnarray*}$$

### 2AP fluorescence assay to measure promoter DNA melting

The fluorescence intensity (excitation at 315 nm and emission 380 nm) of 2AP labeled promoter (200 nM) in reaction buffer A was recorded before and after the addition of 400 nM Rpo41 and 400 nM Mtf1-WT or C-tail mutants in succession. After subtracting background fluorescence from buffer alone, the contribution of Mtf1-WT or mutants in promoter melting was calculated as fold change over the fluorescence of DNA alone (25).

### 2AP fluorescence titrations to determine the *K_d_* of initiating nucleotides

Increasing concentrations of initiating nucleotides were added to a complex of position -1 modified 2AP promoter DNAs (200 nM), Rpo41 (400 nM), and Mtf1-WT or Mtf1-Δ20 (400 nM) at 25°C in reaction buffer A. Fluorescence emission at 380 nm after excitation at 315 nm was recorded. With the human mitochondrial transcription proteins, we used 100 nM LSP or HSP1 promoter modified with 2AP at the –1 position, 200 nM each of TFAM, POLRMT, and TFB2M in reaction buffer B (50 mM Tris acetate, pH 7.5, 50 mM Na-glutamate, 10 mM magnesium acetate, 1 mM DTT and 0.05% Tween-20. The titration data were fit to a hyperbolic equation to estimate the cumulative *K*_d_ of the initiating nucleotides (6, 25).

### 2AP fluorescence assay to measure initial bubble DNA collapse

Promoter DNA (200 nM) labeled with 2AP at position −4 was incubated with Rpo41 (400 nM) at 25°C in reaction buffer A. Mtf1-WT, or Mtf1-Δ12 (200 nM) was added and transcription was initiated by adding a mixture of NTPs (100 μM) and 3′dNTP (250 μM) to stall complexes at positions +7 to +10. The time course of 2AP fluorescence intensity change was measured as described above. The fluorescence intensities were normalized for comparison.

### Radiometric assay to measure RNA priming and runoff RNA synthesis

Transcription reactions were carried out at 25°C using 1 μM Rpo41, 2 μM Mtf1-WT or deletion mutants, and 2 μM of promoter DNA in reaction buffer A. For runoff RNA synthesis, we used 500 μM ATP, UTP, GTP, and 3′dCTP spiked with [γ-^32^P]ATP. The 2-mer synthesis was measured at increasing concentrations of ATP (0-4000 μM) or an equimolar mixture of ATP and GTP. Reactions were stopped after 15 min using 400 mM EDTA and formamide dye (98% formamide, 0.025% bromophenol blue, 10 mM EDTA). Samples were heated to 95°C for 2 min, chilled on ice, and the RNA products were resolved on 24% sequencing gel containing 4 M urea. The gel was exposed to a phosphor screen overnight and scanned on a Typhoon 9410 PhosphorImager instrument (Amersham Biosciences). The free ATP and RNA bands were quantified using ImageQuant and molar amounts of RNA synthesized were calculated according to Equation (2).

(2) $$\begin{equation*}{\rm{RNA}}\,{\rm{synthesized}}\,\,\left( {{\rm{\mu M}}} \right) = \frac{{\rm{R}}}{{{\rm{R}} + {\rm{A}}}}.\left[ {{\rm{ATP}}} \right]\left( {{\rm{\mu M}}} \right)\end{equation*}$$

where R and A are the band intensities of RNA products and free ATP, respectively, and [ATP] is the molar concentration of ATP added to the reaction. The rates of RNA synthesis were divided by the limiting concentration of Rpo41 in the enzyme–DNA complex (1 μM) to obtain the rate constant of RNA synthesis. The rate constants of 2-mer RNA synthesis were plotted as a function of increasing concentration of NTPs, and the curves were fit a hyperbola to obtain the *K*_m_ and *k*_cat_ of 2-mer synthesis (23).

The runoff synthesis reactions with the human mitochondrial transcription proteins were carried out as described above. The catalytic efficiency of RNA priming by TFB2M-WT and deletion mutants was determined similarly as 2-mer synthesis described above with the yeast transcription system. We used 1 μM each of POLRMT, TFAM and TFB2M or TFB2M C-tail deletion mutant and 1 μM of LSP DNA at 25°C, and measured A-ladder RNAs with 0–4000 μM of ATP (spiked with [γ-^32^P] ATP) (6).

### Single-molecule FRET measurement

We used a home-built total internal reflection fluorescence (TIRF) microscope described previously (26) for measuring single-molecule fluorescence signals. The sample surface was prepared by coating quartz slides (Finkenbeiner, USA) with a 40:1 mixture of mPEG-SVA (MW 5000) and biotin-PEG-SVA (MW 5000) (Laysan Bio, USA) after treatment with (3-aminopropyl) trimethoxysilane (Sigma, USA). The surface was coated with NeutrAvidin (ThermoFisher Scientific, USA), and DNA templates were immobilized through biotin-NeutrAvidin interactions. 100 nM of Rpo41 and 100 nM of Mtf1-WT or Mtf1-Δ12 were flowed in the chamber and incubated for 3 min, and excess unbound proteins were washed away. Imaging buffer was added, which contained 100 mM Tris-acetate pH 7.5, 50 mM Potassium glutamate, 10 mM magnesium acetate, 0.6% glucose 1 mg/ml glucose oxidase (from Aspergillus niger VII; Sigma, USA), 0.04 mg/ml catalase (from bovine liver; Sigma, USA), 0.8% dextrose, ∼3 mM Trolox and 500 μM ATP or 500 μM ATP plus 500 μM GTP for stalling the transcription initiation complex at +1 or +2 position, respectively. Fluorescence movies with donor and acceptor channels were recorded using an EMCCD camera (iXon Ultra 897; Oxford Instruments, UK). All measurements were performed at 25°C.

### Single-molecule FRET data analysis

Movies from the TIRF microscope were analyzed by custom software to extract single-molecule fluorescence traces, as described earlier (12). FRET efficiency was calculated with background and leakage correction as *E*_FRET_  =  (*I*_A_ − 0.08  ×  *I*_D_)/(*I*_D_ + *I*_A_), where *I*_D_ and *I*_A_ are the background-subtracted intensities of donor and acceptor dyes, respectively. Acceptor dyes were briefly excited at the beginning and end of each movie to exclude traces lacking acceptor dyes from further analysis. Each FRET histogram was built from >50 movies by selecting the traces with a single pair of Cy3 and Cy5 dyes and representing each trace by *E*_FRET_ averaged over five frames. Hidden Markov analysis was performed by using ebFRET software developed by Gonzalez group (27).

## RESULTS

### Deletion of the Mtf1 C-tail reduces abortive RNA synthesis

**Figure 2.**
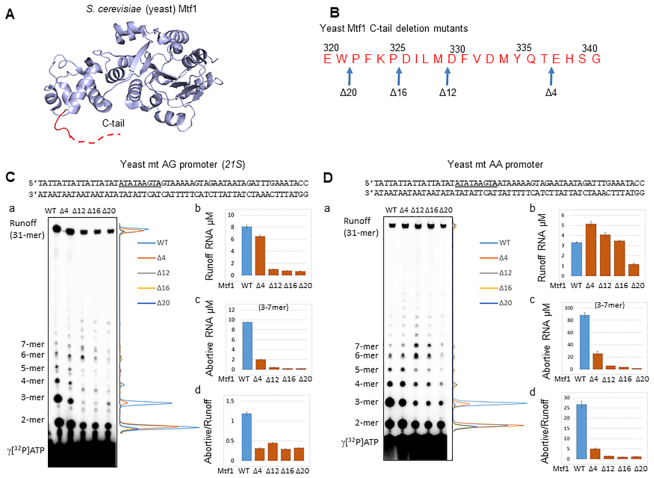
Deletion of the Mtf1 C-tail reduces abortive RNA synthesis. (**A**) Structure of the yeast Mtf1 (PDB: 1I4W) with the missing C-tail region in the red dotted line. (**B**) The amino acid sequence of the yeast Mtf1 C-terminal tail shows the deletion mutants. (**C**, **D**) Sequence of the yeast mitochondrial AG and AA promoters (–25 to +32). The nonanucleotide consensus sequence is underlined and start-site is in bold. (a) Image of the polyacrylamide gel (24% polyacrylamide 4 M urea denaturing gel) and the line graphs show the RNA products of the transcription reactions carried out with 1 μM Rpo41, 2 μM Mtf1 and 2 μM promoter duplex for 15 min using 250 μM ATP, UTP, GTP and 1.25 mM 3′dCTP spiked with γ[^32^P]ATP. Panels b, c, and d show the μM amounts of runoff RNA, 3–7 mer abortive RNA, and abortive to runoff ratio on the AG promoter and AA promoter. Error bars are from two measurements.

To understand the role of Mtf1 C-tail in transcription initiation, we systematically deleted 4–20 amino acids (aa) of Mtf1 from the C-terminus (Figure 2A, B) and purified the C-tail deletion mutant proteins to homogeneity (Supplementary Figure S2A). Since the mitochondrial yeast promoters most commonly initiate with +1+2 AA and AG start-sites (23,28), we tested both types of promoters in transcription runoff assays with the reconstituted transcriptional complex. We used the *21S* rRNA yeast promoter as an example of the AG promoter and changed the +2 G:C to A:T to make the AA promoter. With this design, the rest of the promoter sequence remains identical in both promoters (Figure 2C, D). We also tested the yeast *15S* rRNA promoter fragment that naturally initiates with the AA start site (Supplementary Figure S3).

A typical *in vitro* transcription reaction profile on the promoter fragments consists of an initial distributive phase of abortive products from 2-mer to 7-mer in progressively decreasing amounts, followed by a processive elongation phase when the RNA is elongated to the runoff length (Figure 2C, Da). The Mtf1 C-tail deletion mutants generated the correct 31-mer length runoff product. Hence, C-tail deletion does not alter the start-site of RNA synthesis.

The C-tail deletion, however, reduced the amount of runoff products on the AG promoter (Figure 2 Cb). A defect in runoff synthesis on the AA promoter was not observed until 20 aa of the C-tail were deleted (Figure 2Db, Supplementary Figure S3). Therefore, C-tail deletion has differential effects on the AA and AG promoters. Since the AA and AG promoters have identical upstream and downstream DNA sequences, the higher sensitivity of the AG promoter to C-tail deletion is due to the +2 GC base pair. Previous studies have shown that promoters with +2 non-template adenine transcribe RNA more efficiently than promoters with guanine or thymine (23).

The most striking outcome of C-tail deletion was on the production of short abortive RNAs (Figure 2C, Da and c). The 21S AG promoter generated relatively low amount of abortives in comparison to the AA promoter (Figure 2Cc and 2Dc). This is consistent with previous studies that showed that AA promoters make more abortives than the AG promoters (23). Interestingly, C-tail deletion reduced the amount of short abortives on both promoters (Figure 2C, Da). The 2-mer abortives were decreased by 3–10-fold on the AA and AG promoters, but 3-mer abortives were decreased more drastically (100–300-fold on the AG promoter and 40–100-fold on the AA promoter). The decrease in 2-mer abortives in mutants is due to a defect in transcription initiation, because 2-mer decrease in the mutants was similar to the decrease in runoff product. On the other hand, 3-mer decrease in the mutants was 25–50-fold more than that of the runoff product on both AA and AG promoters. We summed up 3–7 abortive RNAs (Figure 2C, Dc) and compared their amounts to runoff RNA to calculate the abortive to runoff ratio. The abortive to runoff ratio (Figure 2C, Dd) of the C-tail deletion mutants is consistently lower than that of the Mtf1-WT on both AA and AG promoters. This observation supports the model that the Mtf1 C-tail is in the path of the growing RNA and steric clashes between the C-tail and RNA–DNA hybrids result in abortive synthesis. Based on our data, we propose that the Mtf1 C-tail region clashes with the RNA–DNA hybrid starting at 3-mer synthesis.

### Deletion of the Mtf1 C-tail does not affect promoter melting

To understand the role of Mtf1 C-tail in transcription initiation, we assayed each step of transcription initiation. The basic mechanism of transcription initiation is conserved between single-subunit and multisubunit RNAPs, although the details are different. Transcription initiation begins with the binding of RNAP to the promoter DNA to generate a closed complex, followed by DNA melting to form an open complex. The promoter DNA in the open complex of the single-subunit RNAP is severely bent around the melted initiation site (Supplementary Figure S6). After the melted template strand is positioned in the active site, the resulting initiation complex recruits the initiating nucleotides and makes 2-mer RNA to prime the transcription reaction. The 2-mer RNA elongates to a maximum length and triggers a conformational change that releases upstream promoter contacts with the RNAP. Promoter release, coupled with upstream bubble collapse, converts the initiation complex to the elongation complex. We have developed several ensemble radiometric/fluorimetric and single-molecule FRET methods to quantitatively measure each step of transcription initiation through our studies of T7 and yeast mitochondrial RNAPs (12,22,29–32). Here, we employed some of the above techniques to elucidate the functions of the C-tail of Mtf1 and TFB2M.

To determine if the C-tail region is important for the formation of the ternary Rpo41-Mtf1-promoter complex, we measured the *K*_d_ of the complex using fluorescence anisotropy-based titration experiments (Supplementary Figure S2B, C). Rpo41 and Mtf1-WT formed an extremely stable complex with the promoter DNA (0.12 nM *K*_d_) consistent with published results (11). Deletion of 4 and 12 aa of the Mtf1 C-tail did not destabilize the complex, but the deletion of 16 and 20 aa resulted in ∼7-fold weakening of the complex. These results suggest that C-tail interactions are required for stable complex formation between Mtf1 and Rpo41–promoter DNA complex.

**Figure 3.**
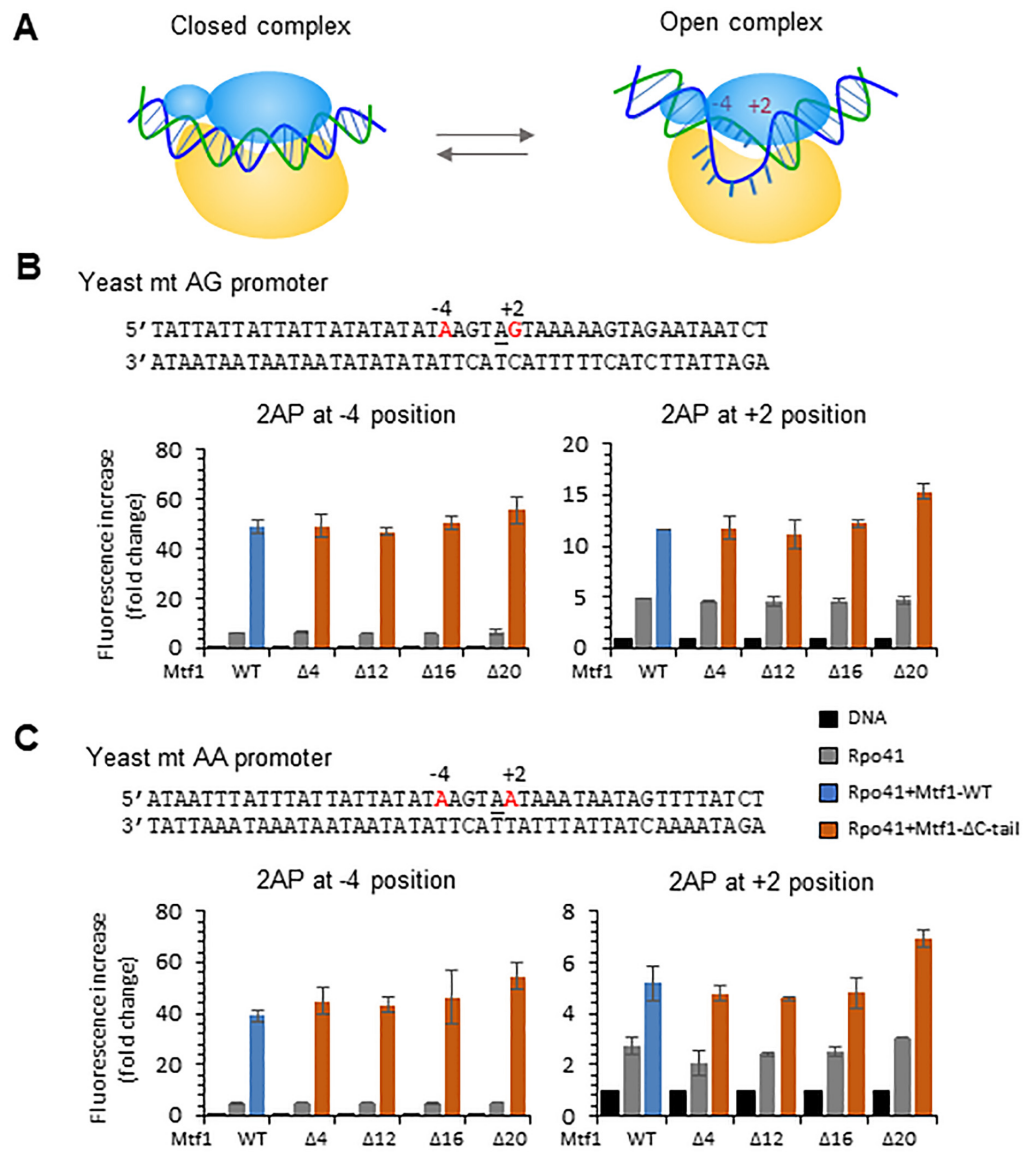
The C-tail of Mtf1 is not essential for promoter DNA melting. (**A**) The cartoon shows the conversion of the closed complex to the bent open complex where the DNA is melted between positions −4 and +2. (**B**, **C**) Sequence of the *21S* rRNA AG promoter (−25 to +20) and 1*5S* rRNA AA promoter (−25 to +20) modified with 2AP (in red) at the −4 or +2 position. The 2AP fluorescence intensity was measured using 200 nM promoter DNA, 400 nM Rpo41 (gray bars) and 400 nM Mtf1-WT (blue bars) or Mtf1 C-tail deletion mutants (orange bars) at 25°C. The bar charts show the fold change in 2AP fluorescence relative to the free DNA. Error bars are from two independent measurements.

To determine if the C-tail region plays a role in promoter melting, we used the 2-aminopurine (2AP) fluorescence-based assay to measure DNA melting with the C-tail deletion mutants (Figure 3A) (25). The 2AP fluorescence is sensitive to base-pairing and base-stacking interactions in duplex DNA (33). When the DNA melts and the 2AP base unstacks, there is a substantial increase in 2AP fluorescence. Using this assay, we showed previously that the yeast and human mitochondrial promoters are melted from −4 to +2 positions in the initiation complex (6,25). We introduced 2AP base individually at −4 and +2 positions representing the upstream and downstream bubble junctions, respectively (Figure 3B, C). The fluorescence intensity of 2AP at position −4 was low in free DNA and increased by 6-fold upon addition of Rpo41 and 40-fold upon addition of Rpo41+Mtf1-WT (Figure 3A, blue bar). Similarly, the fluorescence intensity of 2AP at position +2 in the AG promoter increased by 12-fold upon addition of Rpo41 and Mtf1-WT. Interestingly, none of the C-tail deletion mutants of Mtf1 showed a defect in melting the AG or the AA promoter (Figure 3A, B, red bars). Based on these results, we conclude that the C-tail of Mtf1 is not essential for promoter melting. This is in contrast to a previous study that suggested that the C-tail is necessary for promoter opening (15).

### Deletion of the Mtf1 C-tail causes a severe defect in the binding of initiating nucleotides

**Figure 4.**
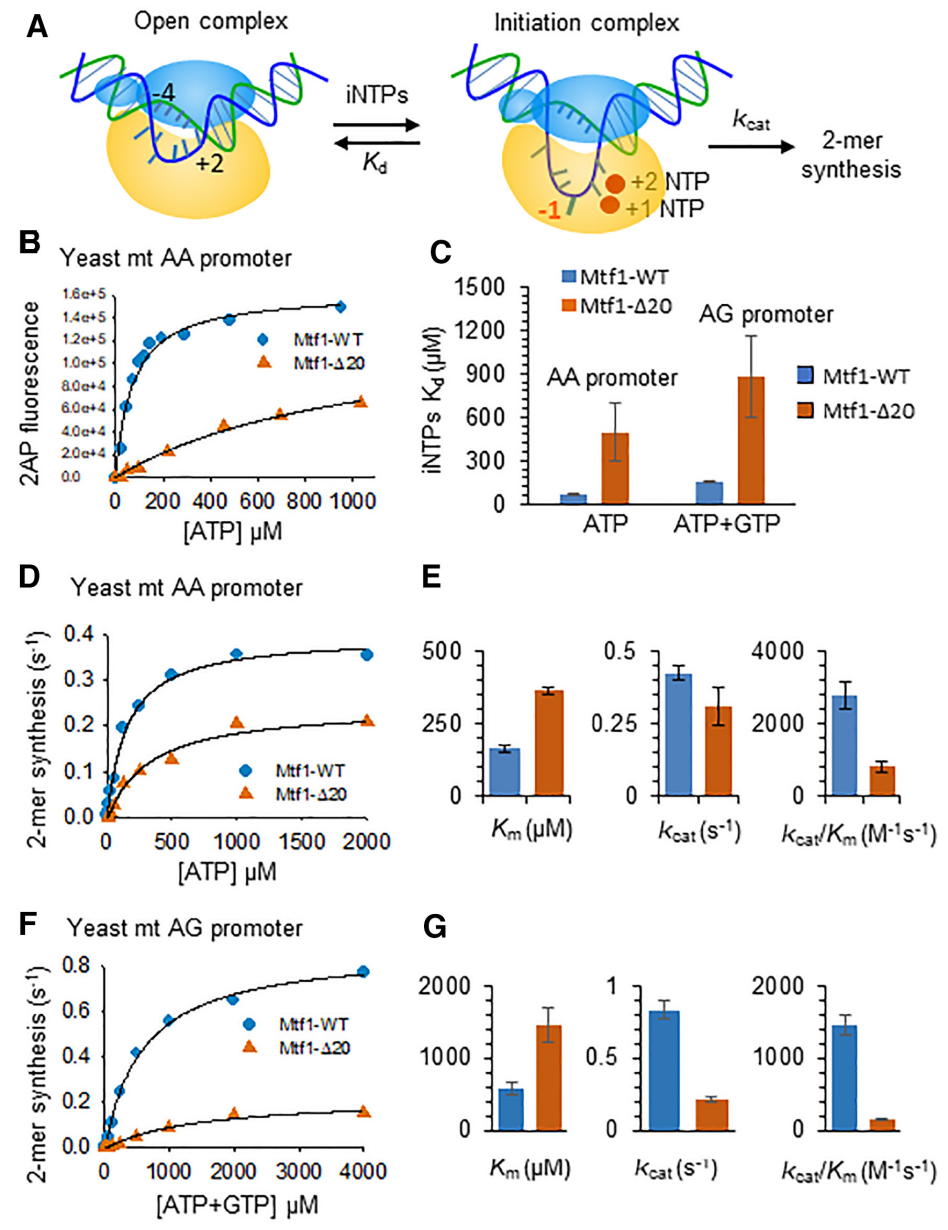
Mtf1 C-tail deletion causes a severe defect in initiating nucleotide-binding and RNA priming. (**A**) The cartoon shows the transition of the open complex to the +2 initiation complex with the template strand aligned in the active site for initiating NTP binding and RNA synthesis. The –1 template base (red) was substituted with 2AP. (**B**) A representative binding curve shows the 2AP fluorescence intensity changes with increasing ATP concentration. The titration experiments were carried out using Rpo41 (400 nM), Mtf1-WT or Mtf1-Δ20 (400 nM) and –1 2AP modified *15S* rRNA AA promoter (200 nM). The data were fit to a hyperbola to obtain the composite *K*_d_ values for the +1+2 initiating NTPs (solid line). Similar titrations were performed on –1 2AP modified *21S* rRNA AG promoter using equimolar mixture of ATP + GTP. (**C**) *K*_d_ values for the +1+2 initiating NTPs are shown in the bar chart. (**D**) 2-mer RNA synthesis was measured as a function of increasing concentrations of ATP on the *21S* rRNA AA promoter (sequence in Figure 3C), and the data were fit to the Michaelis-Menten equation to obtain the *k*_cat_, *K*_m_ and *k*_cat_/*K*_m_ values in (**E**). (**F**) 2-mer RNA synthesis was measured at increasing concentrations of equimolar ATP + GTP on the *21S* rRNA AG promoter (sequence in Figure 3B) and the kinetic constants derived from the fits are shown in (**G**). Error bars are from two independent measurements.

After promoter melting, the template strand must be aligned in the active site to bind the incoming +1 and +2 initiating nucleotides and synthesize a 2-mer RNA that primes the RNA transcript (Figure 4A). A tighter binding affinity of the initiating nucleotides is indicative of efficient template strand alignment in the active site and *vice versa*. Therefore, the efficiency of template strand alignment in the active site can be assessed by quantifying the binding affinity of the initiating nucleotides. The fluorescence intensity of 2AP at position –1 is sensitive to the incoming initiating nucleotides (25). Therefore, 2AP fluorescence intensity changes were used to measure the *K*_d_ of initiating nucleotides through titration experiments.

Rpo41+Mtf1 complex was titrated with increasing concentrations of ATP on the AA promoter and with increasing concentration of the equimolar amounts of ATP+GTP on the AG promoter (Figure 4B). The titration data were fit to a hyperbola to estimate the composite *K*_d_ of the initiating nucleotides (Figure 4B, solid line). ATP binds with a high affinity to the Mtf1-WT complex (65 μM *K*_d_) but shows an 8-fold weaker affinity for the Mtf1-Δ20 complex (500 μM *K*_d_) (Figure 4C). Similar trends were observed on the AG promoter, wherein ATP+GTP binding was 6-fold weaker on the Mtf1-Δ20 complex (880 μM) in comparison to Mtf1-WT (150 μM). Thus, C-tail deletion has a pronounced effect on the binding affinity of the initiating nucleotides, which suggests that the Mtf1 C-tail region is involved in template strand alignment.

### Deletion of the Mtf1 C-tail reduces the catalytic efficiency of the RNA priming reaction

To determine if C-tail deletion affects RNA priming, we measured the rate constant of 2-mer RNA synthesis using the gel-based radiometric assay (6,23). This assay quantifies the *k*_cat_, *K*_m_ and the catalytic efficiency (*k*_cat_/*K*_m_) of 2-mer RNA synthesis. We found that C-tail deletion affects both the *K*_m_ and *k*_cat_ of 2-mer synthesis (Supplementary Table S1). On the AA promoter, 20 aa C-tail deletion decreased the *k*_cat_ of 2-mer synthesis by ∼2-fold (*k*_cat_ of 0.4 s^−1^ and 0.25 s^−1^ for Mtf1-WT and Δ20) and increased the ATP *K*_m_ by 2-fold (*K*_m_ of 165 μM with Mtf1-WT and 364 μM with Mtf1-Δ20) (Figure 4D, E). Thus, C-tail deletion reduces the catalytic efficiency of 2-mer synthesis by 3–4-fold (Figure 4E). This explains the 3-fold reduction in runoff synthesis upon C-tail deletion (Figure 2Dc). Our results are consistent with a previous study that observed a defect in nucleotide *K*_m_ with the Mtf1-Δ17 mutant (15).

On the AG promoter, we observed a 2.5-fold higher *K*_m_ of ATP and GTP due to 20 aa C-tail deletion and 4-fold lower *k*_cat_ of 2-mer synthesis (Figure 4F, G). Hence, C-tail deletion reduces the catalytic efficiency of 2-mer synthesis on the AG promoter by 10-fold (Figure 4G), which is consistent with the more significant defect in runoff synthesis on the AG promoter (Figure 2Cc). Mtf1-Δ12 mutant is similarly defective in 2-mer RNA synthesis as the Mtf1-Δ20 mutant (Supplementary Figure S4). Overall, the above studies show that the Mtf1 C-tail region is not essential for promoter melting, but it plays a critical role in template strand alignment in the active site of the initiation complex.

### The C-tail of human TFB2M has a similar role in initiation as the Mtf1 C-tail

**Figure 5.**
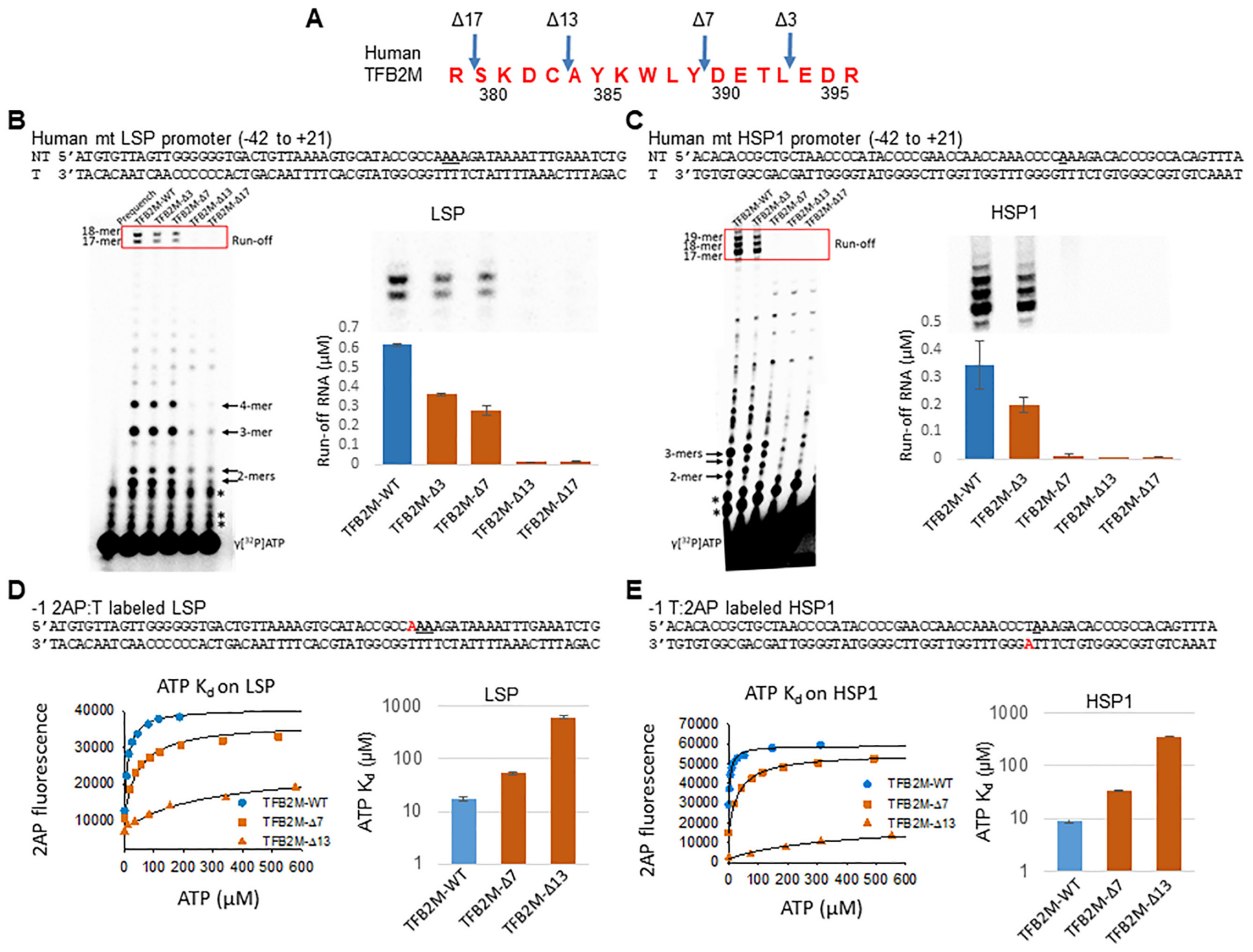
The C-tail region of TFB2M is essential for transcription initiation. (**A**) The amino acid sequence of the human TFB2M C-tail region is shown with the positions of the deletions. (**B**) and (**C**) The sequence of the promoter fragments and the transcription profiles of TFB2M-WT and C-tail deletion mutants on the LSP promoter (B) and the HSP1 promoter (B). The gel image shows the RNA products on LSP (B) and HSP1 (C) fragments. Reactions were carried out with 1 μM each of POLRMT, TFB2M, TFAM, and promoter duplex and 250 μM ATP, UTP, GTP for LSP or ATP, GTP, CTP for HSP1 and γ[^32^P]ATP for 15 min at 25°C. The * represents impurities in the γ[^32^P]ATP also present in the lane without reaction (prequench). The bar charts show μM amount of run-off products boxed in red and also shown above the bar charts. Error bars are from two independent measurements. (**D** and **E**) Binding of the initiating nucleotides with -1 2AP labeled LSP and HSP1 promoters (2AP label shown in red) carried out by titrating 100 nM promoter DNA, 200 nM each of POLRMT, TFAM, TFB2M-WT or the indicated C-tail deletion mutants with increasing amounts of ATP. The binding data were fit to a hyperbola (solid line) to obtain the *K*_d_ values plotted in the bar charts. Error bars are from two independent measurements.

To determine if the C-tail region of the human TFB2M has a similar role in transcription initiation as the yeast Mtf1, we prepared four TFB2M C-tail deletion mutants by deleting 3–17 aa from the C-terminus (Figure 5A, Supplementary Figure S1). Transcription activity was measured with POLRMT, TFB2M and TFAM on the two human mitochondrial promoter fragments with LSP (Light Strand Promoter) and HSP1 (Heavy Strand Promoter 1) sequences (Figure 5B, C). Runoff synthesis on the LSP fragment produces 2–4 mer abortive RNAs and two runoff products, 17- and 18-mer in length (Figure 5B). The two runoff products result from the two reported start-sites on LSP (underlined in the sequence) (6,34). Transcription begins from a single start-site on HSP1, which results in the 17-mer runoff product, while the longer RNAs are derived from slippage synthesis characteristics for this promoter (Figure 5C).

Deletion of 3 and 7 aa of the TFB2M C-tail region decreased runoff synthesis by 2–3-fold on LSP, but deletion of 13 and 17 aa abolished runoff synthesis (Figure 5B). Similarly, deletion of 3 aa of the TFB2M C-tail decreased runoff synthesis by 2-fold on HSP1, but deletion of 7 aa decreased runoff synthesis drastically by 30-fold. Thus, interestingly HSP1 is more dependent on C-tail interactions than LSP. Unlike Mtf1, C-tail deletions in TFB2M showed no noticeable decrease in abortive RNAs.

To determine if the C-tail region of TFB2M plays a role in template strand alignment, we measured the *K*_d_ of the initiating ATPs using fluorescence-based titrations with promoters labeled with 2AP at position −1 (Figure 5D and E). C-tail deletions in TFB2M had a large effect on the binding affinity of the initiating ATP. Deletion of 7 aa decreased ATP binding affinity by 3–4 fold on both promoters and deletion of 13 aa reduced the ATP binding affinity more drastically by 36–40-fold (Figure 5D and E). Thus, the C-tail region in human TFB2M, similar to the yeast Mtf1, is necessary for high-affinity binding of the initiating nucleotides. Interestingly, even though the initiating ATP *K*_d_ value of the TFB2M-Δ7 complex is 30 μM, we see no runoff synthesis with this mutant at 250 μM NTPs (Figure 5C). However, TFB2M-Δ7 does make short RNA products, which suggests that TFB2M C-tail deletion affects the transition from initiation to elongation.

Next, we measured the catalytic efficiency of RNA priming by carrying out transcription reactions with ATP alone. Because the initiation sequence of the promoters begins with +1AAA, transcription reactions with ATP result in A-ladder synthesis. We, therefore, measured A-ladder synthesis as a function of ATP concentration to determine the catalytic efficiency (*k*_cat_/*K*_m_) of RNA priming. The data shows that increasing C-tail deletion leads to an increasing defect in the catalytic efficiency of A-ladder synthesis on the LSP (Supplementary Figure S5). Consistent with runoff synthesis, 3–7 aa deletion of the TFB2M C-tail show a mild effect, but deletion of 13–17 aa reduce RNA priming by 7–30-fold.

The above studies demonstrate that the C-tail regions of Mtf1 and TFB2M have similar roles in enabling efficient binding of the initiating nucleotides and promoting the RNA priming reaction. We propose that these functions are supported by the C-tail region due to its ability to position the template strand in the active site of the initiation complex.

### Early initiation complexes exhibit conformational disorder with C-tail-deleted Mtf1

**Figure 6.**
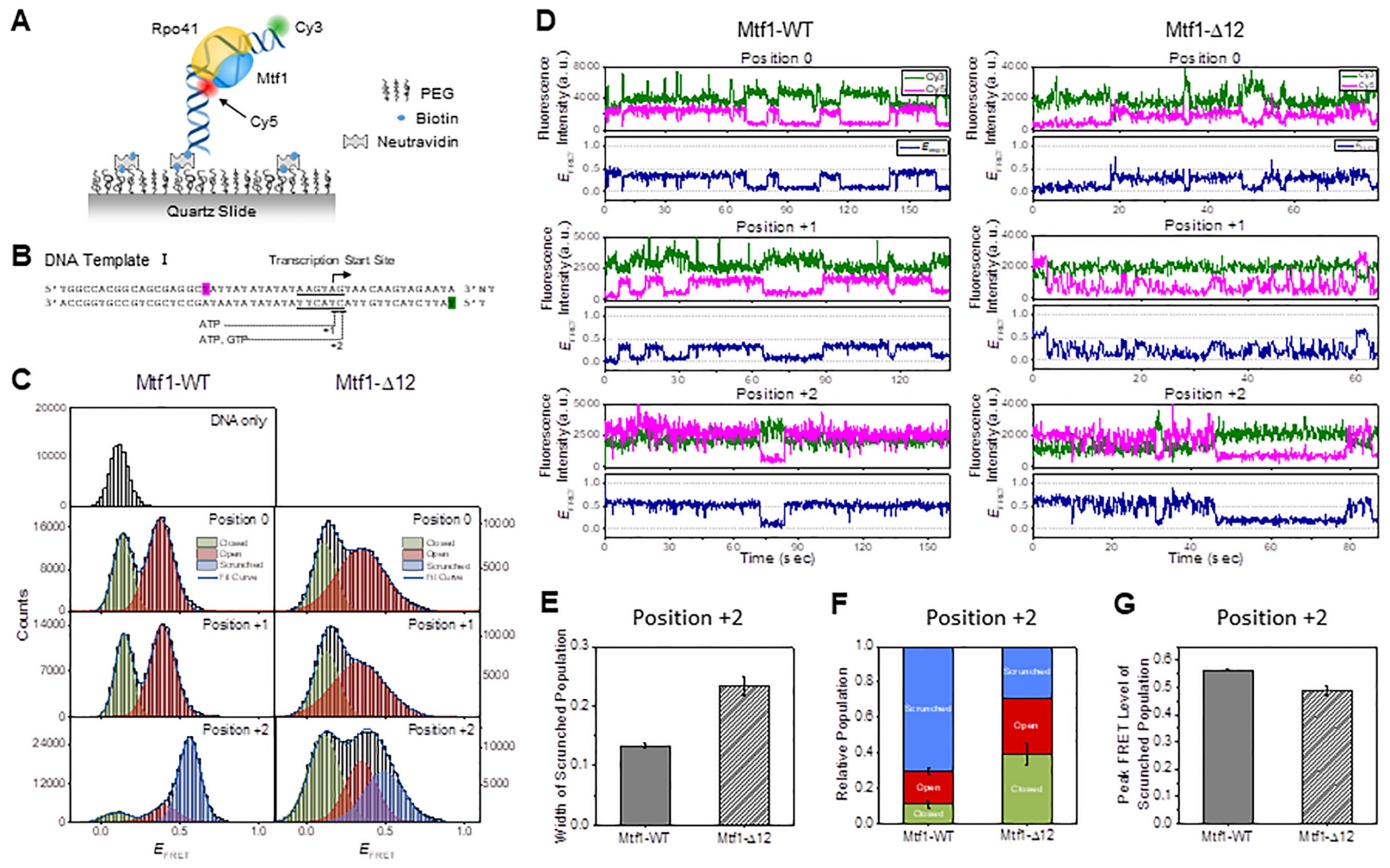
Single-molecule FRET time traces measure the dynamics of open and initiation complexes of Mtf1-WT and Mtf1-Δ12. (**A**) Schematic representation of the single-molecule set up to measure the dynamics of the transcription initiation reaction. Dual-labeled promoter DNA in complex with Rpo41 and Mtf1 is observed under a total internal reflection fluorescence microscope. (**B**) The sequence of the DNA template used in single-molecule FRET measurements. Dye labeling positions are highlighted in magenta (Cy5) and green (Cy3). Consensus nonanucleotide promoter sequence is underlined, and the transcription start site is marked with an arrow. The initiation complex can be stalled at +1 or +2 position by supplying 0.5 mM ATP or ATP + GTP. (**C**) FRET histograms from single-molecule FRET time traces with co-localized Cy3 and Cy5 signals at each stalling position for Mtf1-WT and Mtf1-Δ12. Histograms were fit to two or three Gaussian peaks. Green, red, and blue curves represent closed, open, and scrunched populations, respectively. (**D**) Representative single-molecule FRET time traces at positions 0, +1 and +2, shown for Mtf1-WT and Mtf1-Δ12. Cy3 (green) and Cy5 (magenta) signals and calculated FRET efficiency (navy) are shown. (**E**) Gaussian width of the scrunched population at position +2, compared between Mtf1-WT and Mtf1-Δ12. (**F**) The relative population of closed, open, and scrunched conformations at position +2, compared between Mtf1-WT and Mtf1-Δ12. (**G**) Mean FRET level of the scrunched population at position +2, compared between Mtf1-WT and Mtf1-Δ12.

Previously, we had reported that single-molecule FRET measurements could be used to study the dynamics of promoter opening and closing steps through promoter bending/unbending by Rpo41-Mtf1 (12). To trace the conformational dynamics of the promoter DNA complexed with Rpo41 + Mtf1, we immobilized the promoter DNA on a surface and formed a complex with free proteins (Figure 6A). The 50 bp AG promoter DNA was labeled with Cy5 dye at position -16 of the non-template strand and with Cy3 dye at position +16 of the template strand (Figure 6B). In this configuration, FRET efficiency between the fluorophores reports the bending/unbending transition in the open complex and scrunching/unscrunching motions of the promoter DNA during RNA synthesis in the initiation stage. To initiate 2-mer RNA synthesis, the RNAP unwinds the downstream DNA and brings the +1 and +2 position templating bases into the active site for pairing with the initiating NTPs. During this process, the upstream promoter contacts are stably maintained; hence, the transcription bubble does not expand, and instead, the initially melted −4 to −1 template nucleotides scrunch to accommodate the RNA–DNA hybrid, and this is referred to as DNA scrunching. Unscrunching is the reverse process where the +2 complex changes back to the open complex.

First, we collected single-molecule time traces with Mtf1-WT in the absence of initiating nucleotides at position 0. The histogram of FRET efficiency constructed from single-molecule time traces of the DNA template alone showed a single peak at a low-FRET level, *E*_FRET_ = 0.14 (Figure 6C). Upon adding Rpo41 and Mtf1-WT, we observed an additional peak at a mid-FRET level, *E*_FRET_ = 0.38. The single-molecule time traces showed reversible transitions between the low- and mid-FRET states (Figure 6D). Because FRET was measured after washing free unbound proteins away, and histograms were built from traces that visited the mid-FRET level at least once, these reversible FRET transitions represent equilibrium motions within the complex rather than arising from dissociation and rebinding of proteins. Such dynamics are consistent with the previously observed promoter opening-closing transitions (12,22), and we assigned the low-FRET level to a closed promoter state and the mid-FRET level to an open promoter state.

FRET dynamics and histogram with Rpo41+Mtf1-WT at position +1 with 0.5 mM ATP did not show a significant difference from those at position 0, implying that +1 ATP does not significantly affect the structure of the open complex (Figure 6C, D). It is possible that +1 ATP in the absence of +2 GTP dissociates from the complex quickly and therefore has little effect on the opening-closing transitions. On the other hand, FRET histogram at position +2 with 0.5 mM each of ATP and GTP showed a marked appearance of a new dominant population at a higher FRET level, *E*_FRET_ = 0.56, leaving only small populations at the previous FRET levels (Figure 6C), consistent with the observation in a recent work (22). The higher FRET state represents a scrunched/bent template conformation, which is a dominant state at position +2 with Mtf1-WT. Interestingly, the scrunched/bent conformation reversibly transitioned to the mid- or low-FRET states, as evidenced by frequent FRET transitions in the single-molecule time traces (Figure 6D).

We used Mtf1-Δ12 as a representative C-tail mutant in the single-molecule studies because it forms a stable complex with Rpo41 in comparison to the Mtf1-Δ20 mutant. With Mtf1-Δ12 at position 0, we observed a mid-FRET level at *E*_FRET_ = 0.38 as with Mtf1-WT; however, in contrast to Mtf1-WT, the mid-FRET population showed a broader distribution with Mtf1-Δ12 (Figure 6C). The FRET traces with Mtf1-Δ12 exhibited reversible switching between low- and mid-FRET states, and consistent with the wide FRET distribution, they showed rapid fluctuations (Figure 6D). The FRET histogram at position +1 was similar to that at position 0. The FRET histogram at position +2 was also broad and showed an increase in population at a higher FRET level (Figure 6C). As the FRET traces at this position exhibited a distinguishable higher FRET state that frequently transitioned to and from the mid-FRET state (Figure 6D), we fit the histogram to three Gaussian peaks keeping two FRET levels the same as those found at position +1. This analysis revealed a new FRET population at *E*_FRET_ = 0.50, which is broader than that with Mtf1-WT (Figure 6C, E).

Thus, single-molecule FRET measurements indicate that Rpo41 and Mtf1-Δ12 can form an open promoter complex, but the broad FRET distribution and rapid fluctuations suggest the existence of a conformational ensemble and greater promoter flexibility in the mutant complex in comparison to the complex with Mtf1-WT. These results also indicate that the scrunched/bent initiation complex at position +2 with Mtf1-Δ12 has a lower stability as demonstrated by the decreased population of the high FRET (scrunched) state and shorter lifetime relative to the complex with Mtf1-WT (Figure 6D, F). The complexes at position +2 with Mtf1-Δ12 showed a slightly lower FRET level than that with Mtf1-WT, which indicates that it has a different structure, probably less scrunched/bent than that with Mtf1-WT (Figure 6G).

**Figure 7.**
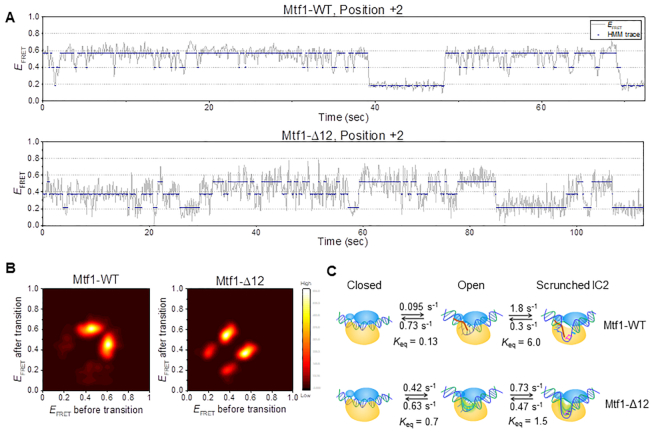
Hidden Markov analysis of single-molecule FRET time traces reveals the rates of transition between closed, open, and scrunched initiation complexes for Mtf1-WT and Mtf1-Δ12. (**A**) Representative single-molecule FRET time traces (gray) at position +2, shown along with hidden state traces (navy) from hidden Markov modeling assuming three states. (**B**) Transition density plots from hidden Markov analysis of FRET traces at position +2. 184 and 175 traces were used for Mtf1-WT and Mtf1-Δ12, respectively. (**C**) Kinetic model of transcription initiation based on the results from hidden Markov analysis. Mtf1 is shown in blue with C-tail in red and Rpo41 in yellow. The template DNA is colored blue and non-template in green. Transition rates between closed, open, and scrunched initiation conformations are compared between Mtf1-WT and Mtf1-Δ12.

To uncover the dynamic behavior of the +2 initiation complex, we performed a hidden Markov analysis of the FRET time traces, assuming three hidden states, representing closed, open, and scrunched states (Figure 7A). The transition density plot of the +2 initiation complex with Mtf1-WT was dominated by scrunching-unscrunching transitions whereas the one with Mtf1-Δ12 showed a substantial fraction of opening-closing transitions (Figure 7B). A comparison of the transition rate constants (Figure 7C, Supplementary Table S2) revealed a faster transition to the open complex with Mtf1-Δ12 compared to Mtf1-WT. However, Mtf1-Δ12 showed a lower scrunching rate and a higher unscrunching rate of the +2 initiation complex relative to Mtf1-WT (*K*_eq_ of 1.5 *vs*. 6 for Mtf1-Δ12 *vs*. Mtf1-WT), which indicates reduced stability of the scrunched conformation with Mtf1-Δ12. Thus, ensemble and single-molecule FRET measurements consistently suggest that the C-tail of Mtf1 is necessary for stabilizing the scrunched/bent conformation of the +2 initiation complex with the template productively aligned in the active site for RNA priming.

### The C-tail of Mtf1 is crucial for the initially transcribing complexes to progress to the elongation stage

**Figure 8.**
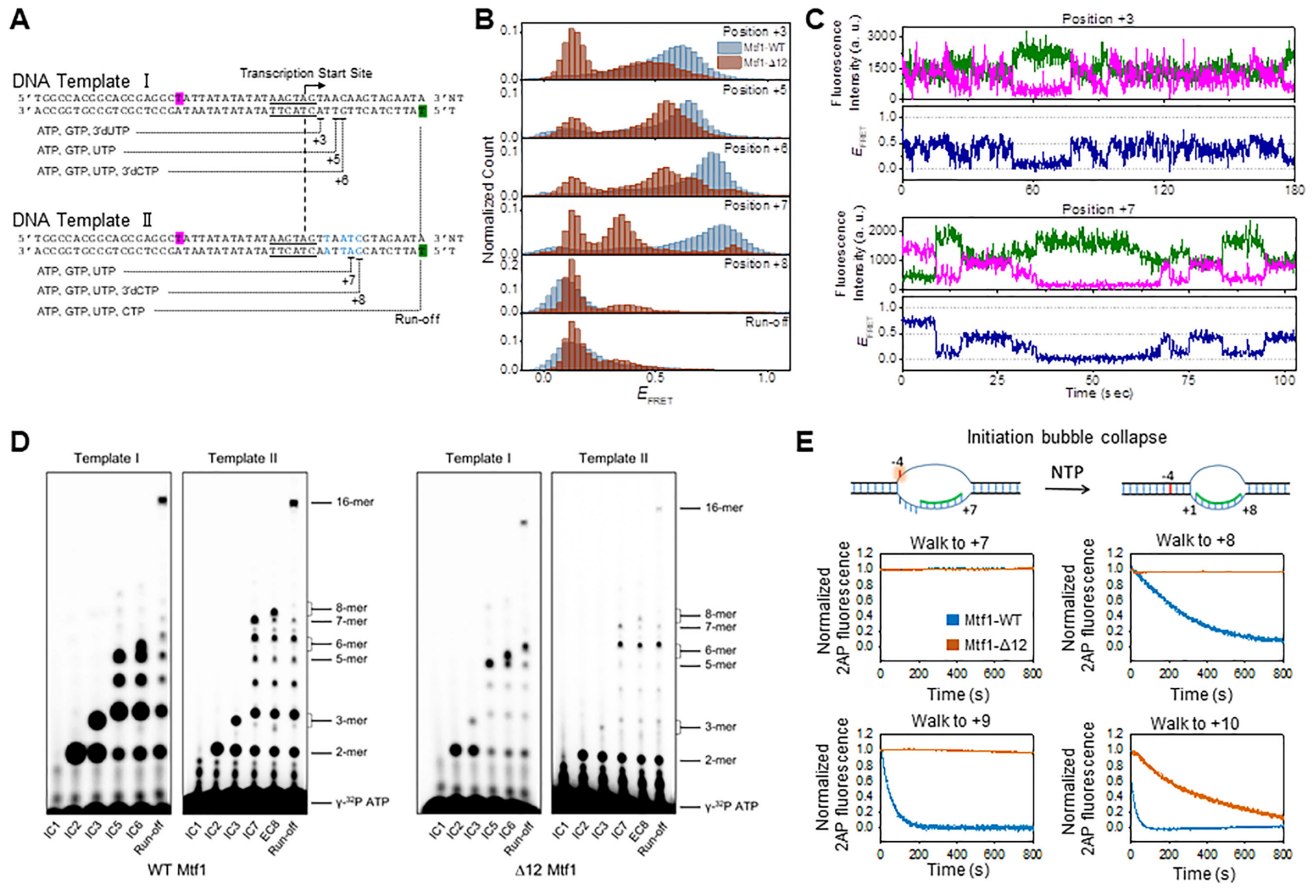
Mtf1 C-tail is crucial for stabilizing the scrunched initiation complexes to progress to the elongation stage. (**A**) Schematic design of DNA templates used in transcription walking experiments. DNA template I can be stalled at positions +3, +5 and +6. DNA template II is different from DNA template I by four base-pairs (blue) and stalls transcription at positions +7 and +8 with different combinations of 0.5 mM each of the nucleotide substrates. Both templates were labeled at position −16 of the non-template strand with Cy5 (highlighted in magenta) and position +16 of template strand with Cy3 (highlighted in green). Transcription promoter (underlined) and start site (arrow) are marked. (**B**) FRET histograms at each stalling position measured with Mtf1-WT (blue) and Mtf1-Δ12 (brown). (**C**) Representative FRET time traces with Mtf1-Δ12 at positions +3 and +7. (**D**) RNA products from transcription reaction on DNA templates I and II. Transcription reactions were carried out with 2 μM each of Rpo41, Mtf1, and DNA and indicated NTPs in (A) at 100 μM, each spiked with γ[^32^P]ATP. Double band locations were observed for 3-mer, 6-mer and 8-mer products due to differential migration of NTP and 3′-dNTP. (**E**) The cartoon shows the 2AP at position −4 in the +7 initiation complex in an unstacked high fluorescence intensity state and annealed in a duplex in a low fluorescence intensity state in the +8 elongation complex. DNA templates III and IV were modified with 2AP at position −4 (Supplementary Figure S9). The experiment was performed with 200 nM DNA and 400 nM each of Rpo41 and Mtf1-WT or Mtf1-Δ12. Transcription reactions were stalled at positions between +7 and +10 by adding combinations of NTPs (100 μM) and 3′dNTP (250 μM), and representative time traces of repeats of 2AP fluorescence are shown.

Next, we advanced the +2 initiation complex to later stages of transcription by stalling RNA synthesis at positions +3, +5, +6, +7 and +8 using different combinations of nucleotides on DNA templates I and II (Figure 8A). Mtf1-WT complex catalyzes RNA elongation by the DNA scrunching mechanism, wherein the initially melted template region and newly melted non-template region scrunches to accommodate the growing RNA–DNA hybrid (22). Due to DNA scrunching/bending, the distance between the two fluorophores at positions −16 and +16 in the promoter DNA progressively decreases with each nucleotide addition, which is observed as FRET increases. This was evident in Mtf1-WT, where we observed a progressive increase in FRET level with each nucleotide addition from 2-mer to 6-mer RNA synthesis (*E*_FRET_ ∼ 0.6 to 0.75) (Figure 8B, blue-shaded histograms from Sohn *et al.* (22)). By contrast, at each stalling position up to +6, Mtf1-Δ12 showed broader FRET distributions, a smaller percentage of WT-like high FRET level complexes, and a significant portion of lower FRET level complexes (*E*_FRET_ ∼ 0.55) (Figure 8B). Upon walking to position +7, Mtf1-WT progressed to an even higher FRET level (*E*_FRET_ ∼ 0.8) than that at position +6. On the other hand, the FRET level decreased (*E*_FRET_ ∼ 0.35), which is close to the mid-FRET level observed at position 0 (*E*_FRET_ ∼ 0.38), whereas the higher FRET level in the Mtf1-Δ12 was a minor population (Figure 8B).

Single-molecule FRET traces of Mtf1-Δ12 at position +3 exhibited fast fluctuations in the high FRET range, suggesting that the broad FRET distribution in +3 stalled complexes is due to rapid conformational changes, similar to the observations at positions +1 and +2 (Figure 8C). Single-molecule FRET traces at position +7 rarely showed transition to the high-FRET state and switched mostly between low- and mid-FRET states (Figure 8C). Overall, the lower FRET levels in the Mtf1-Δ12 complexes suggest less and rarer highly scrunched/bent DNA states during the initiation stage. We conclude that the Mtf1 C-tail region is essential for supporting the DNA scrunching mechanism during transcription initiation.

A hallmark of transition from initiation to elongation is the release of the promoter contacts and upstream bubble collapse. Transition to elongation typically occurs between 8–12 nt RNA synthesis (31,35). The promoter DNA in the initiation complexes is highly bent, and when the upstream promoter is released, it assumes a less bent conformation in the elongation complex (Supplementary Figure S6) (13,36). Previous studies with Mtf1-WT showed that upon walking to position +8, the FRET level suddenly dropped to a low FRET level, consistent with the transition into elongation (Figure 8B) (22). With Mtf1-Δ12, the mid- or high-FRET population also decreased to lower FRET level at position +8. Because the closed complex is indistinguishable from the elongation complex on the −16/+16 labeled DNA, we used another DNA template with Cy3 and Cy5 attached at positions +11 and −11 of template strand and non-template strand, respectively, which can distinguish between closed complex and elongation complex at position +8 (Supplementary Figure S7A). When stalled at position +8 on this DNA template, the primary FRET level with Mtf1-WT was distinct from both the closed DNA and the +7 initiation complex (Supplementary Figure S7B). In contrast, the FRET level of the +11/−11 DNA at position +8 with Mtf1-Δ12 was similar to that of the closed DNA. This suggests that Mtf1-Δ12 does not form the WT-like elongation complex. Instead, the initiation complex of Mtf1-Δ12 at position +8 returns to the closed promoter state by abortive dissociation of the RNA transcript or is stalled in a low FRET conformation with the RNA transcript bound to the template DNA. Such a contrasting behavior of Mtf1-WT and Mtf1-Δ12 at positions +7 and +8 was consistently observed in another DNA template with a different sequence in the initiation region (Supplementary Figure S8). Overall, our data suggest that Mtf1-Δ12 forms structurally different intermediate states along the initiation pathway from those with Mtf1-WT, making it less capable of progressing to elongation.

Next, we carried out transcription reactions on DNA Templates I and II using NTP mixes as in the above single-molecule FRET measurements and analyzed the RNA products on polyacrylamide gels with single-nucleotide resolution (Figure 8D). With Mtf1-WT, we observed the correct maximum length transcript expected at each stalling position and abundant abortive transcripts. Transcription with Mtf1-Δ12 also showed the correct maximum length transcript up to position +6 and lower amounts of abortive products beyond 2-mer, consistent with the results in Figure 2D. Upon walking to +7 and +8 positions, Mtf1-Δ12 showed only small amounts of maximum length 7-mer and 8-mer products, and most of the transcription reaction was terminated with 6-mer transcript. Similarly, under run-off condition, 6-mer product accumulated in Mtf1-Δ12 reactions on both Template I and II. These results suggest that Mtf1-Δ12 is defective in forming the highly scrunched/bent position +7 complex because it has problems in elongating the 6-mer RNA to 7-mer. Perhaps the 6-bp RNA–DNA hybrid comes close to the partial C-tail region in Mtf1-Δ12 and the 6-mer RNA aborts. Alternatively, the RNA transcript may remain bound to the template DNA in a stalled low FRET state, such as a backtracked state, suggested recently for Rpo41-Mtf1 (22).

When the initiation complex transitions to the elongation complex, the initially melted −4 to −1 bases reanneal and this is referred to as the upstream bubble collapse. This process can be monitored in real-time using promoter DNA labeled with 2AP at position −4. The upstream bubble collapse results in the decrease of 2AP fluorescence intensity, and the kinetics provides a measure of the efficiency or speed of bubble collapse at each stalling position (Figure 8E). Transcription was stalled at positions +7, +8, +9 and +10 using DNA templates III and IV (Figure 8E, Supplementary Figure S9). At position +7, 2AP fluorescence remained high in both Mtf1-WT and Mtf1-Δ12 complexes, indicating that the −4 base has not reannealed in the +7 stalled complexes. At position +8, Mtf1-WT showed slow kinetics of bubble collapse, which increased in rate when the complex was stalled at positions +9 and +10. This indicates that the upstream bubble collapse initiates in Mtf1-WT complexes after 8-mer RNA synthesis. By contrast, at position +8, Mtf1-Δ12 did not show any sign of bubble collapse, and bubble collapse was observed at position +10. These results indicate that C-tail deletion affects the timing of the transition of the initiation complex to the elongation complex. Additionally, these data indicate that the Mtf1-Δ12 complex stalled at position +8 is not a closed complex. Thus, the low FRET level Mtf1-Δ12 complex at position +8 is a less scrunched/bent initiation complex.

## DISCUSSION

The human and yeast mitochondrial RNAPs rely on transcription initiation factors, Mtf1 and TFB2M, respectively, for promoter-specific transcription initiation. These initiation factors are well known for their essential roles in promoter melting wherein the factors stabilize the open complex by trapping the non-template strand in the nucleic acid binding pocket (6,7,10,13). However, beyond promoter melting, very little was known about their functions in transcription initiation and RNA elongation. In the present study, we have investigated the role of the flexible C-terminal tail regions of Mtf1 and TFB2M proteins. We find that the C-tail region plays a critical role in aligning the template strand in the active site and enabling high-affinity binding of initiating nucleotides for efficient RNA priming reaction. This role of template strand alignment is conserved between Mtf1 and TFB2M. Despite these similarities, we noted that there are differences between TFB2M and Mtf1 in terms of their dependencies on C-tail interactions for runoff synthesis. For example, 13 aa deletion of the C-tail in TFB2M abolished runoff synthesis but a similarly large 20 aa deletion of the Mtf1 C-tail did not abolish but only decreased runoff synthesis. This implies that TFB2M is critically dependent on C-tail interactions for transcription initiation. The exact reason for the different outcomes of C-tail deletion in Mtf1 and TFB2M is not understood. One possibility is that TFB2M may need the C-tail interactions to form a stable complex with POLRMT, whereas Mtf1 may have compensatory ways to bind Rpo41. Nevertheless, additional studies are needed to test these hypotheses.

To investigate the role of Mtf1 C-tail interactions in steps beyond template strand alignment and 2-mer RNA synthesis, we employed a combination of single-molecule FRET and ensemble 2AP based bubble collapse assays. Our recent single-molecule FRET studies characterized each step of the transcription initiation pathway and transition into elongation by Rpo41 and Mtf1-WT (22). These studies showed that Rpo41-Mtf1 catalyzes RNA growth during transcription initiation by the DNA scrunching mechanism, and these studies defined the stage when promoter release and transition into elongation occurs in Mtf1-WT. In the present study, we characterized the initiation pathway of Mtf1-Δ12 and compared it to Mtf1-WT, which shows that C-tail interactions are critical for stabilizing the scrunched DNA conformation in the initiation complexes. Moreover, we found that the C-tail region serves as a sensor of RNA–DNA hybrid length and brings about a timely transition into the elongation. Lacking such detailed studies of the TFB2M, it remains to be determined whether the C-tail of TFB2M has similar functions during transcription initiation.

**Figure 9.**
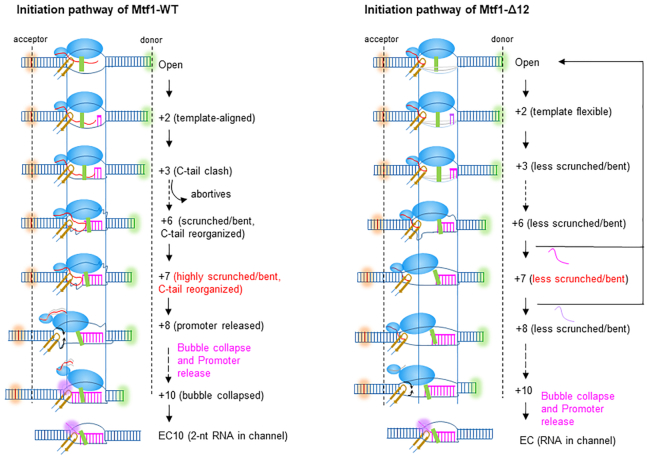
Transcription initiation pathway of Mtf1-WT and Mtf1-Δ12 C-tail deletion mutant. The promoter DNA is typically bent (Figure 1 and Supplementary Figure S8), but in this schematic, the DNA in a linear form shows more clearly the distance changes between the FRET fluorophores with DNA scrunching. The structural features in the model are guided by the TFB2M structure in the open complex of POLRMT (6ERP) (13). The large N-terminal domain of Mtf1 (in blue) binds the melted non-template strand and the small C-terminal domain contacts the –5 to –7 upstream promoter region. The C-tail region (in red) of Mtf1-WT interacts with the intercalating β-hairpin (in yellow) and the thumb domain (in green) as shown in the TFB2M structure (Supplementary Figure S1). The shorter C-tail region of Mtf1-Δ12 interacts with the intercalating β-hairpin but not long enough to engage the thumb domain. The nascent RNA–DNA hybrid is shown in pink and the scrunched template and non-template DNA as squiggly lines. The solid vertical lines mark the distance between upstream and downstream junctions of the transcription bubble, which remains constant during RNA elongation due to DNA scrunching. Thus, the distance between the fluorophores at –16 and +16 positions (dotted lines) decreases with RNA elongation in Mtf1-WT complexes but not in Mtf1-Δ12 complexes. Mtf1-WT complexes make abortive RNAs due to clashes with the RNA–DNA that initiates with 3-bp RNA–DNA. The longer RNA–DNA hybrid resists and reorganizes the C-tail region in Mtf1-WT. At position +8, the RNA–DNA pushes out the C-tail region and the C-terminal domain begins to dissociate followed by an upstream bubble collapse between +8 and +10. The C-tail in Mtf1-Δ12 is too short to clash with the RNA–DNA hybrid hence abortives are sparse. Position +6 and +7 initiation complexes of Mtf1-Δ12 are unstable and abort the RNA or remain bound in less scrunched/bent states. Lack of steric clashes between C-tail and RNA–DNA in Mtf1-Δ12 complexes keeps the C-terminal domain contacts intact and bubble collapse does not begin until +10. The 5′-end of the RNA transcript is threaded into the RNA channel (pink shaded) in the elongation complex (EC) with 8-bp RNA–DNA.

Figure 9 summarizes the detailed transcription initiation pathways of Mtf1-WT and Mtf1-Δ12. The high resolution structure of Rpo41-Mtf1 is not known yet; thus, the structural details of the open complex intermediate of Rpo41 and Mtf1 are guided by the structure of the human POLRMT and TFB2M open complex (13). In the open complex, the promoter DNA is severely bent, as shown in Supplementary Figure S8, but the model in Figure 9 illustrates the DNA in a linear form to highlight distance changes coupled to DNA scrunching. The large N-terminal domain of Mtf1 binds to the melted non-template strand, and the small C-terminal domain interacts with the upstream -7 promoter region. The C-tail region emerging from the C-terminal domain is projected toward the active site in the path of the nascent RNA–DNA hybrid. Like TFB2M, the Mtf1 C-tail is proposed to interact with the intercalating β-hairpin and the thumb domain of the RNAP (Supplementary Figure S1). The intercalating β-hairpin stabilizes the upstream bubble junction, and the thumb domain stabilizes the RNA–DNA hybrid (36). The shorter C-tail region of the Mtf1-Δ12 is shown to interact with the intercalating β-hairpin but not the thumb domain.

Although previous studies suggested that the C-tail region is important for promoter melting (15), direct measurements of base-pair melting by 2AP fluorescence and promoter conformational changes by single-molecule FRET studies show that Mtf1 C-tail is not required for promoter melting. Instead, our data show that the primary role of the C-tail region is to stabilize the initiation complex and the template DNA in the active site. Our single-molecule FRET studies show that C-tail region deletion increases the flexibility of the promoter DNA and hence the transcribing initiation complexes assume various conformational states. Due to this flexibility, the C-tail deletion mutants show weak binding of the initiating nucleotides and slower rates of RNA priming. As indicated above, the template alignment function of the C-tail region is conserved between the yeast and human mitochondrial initiation factors.

After 2-mer synthesis, the RNAP continues to unwind the downstream DNA to bring the template DNA into the active site for RNA elongation. The RNAP maintains upstream promoter contacts throughout initiation; therefore, the initially melted template DNA and the newly unwound non-template DNA gets scrunched. Evidence for the DNA scrunching mechanism has been presented for T7 RNAP and multisubunit RNAPs (16,32,37–39). The evidence for DNA scrunching by Rpo41-Mtf1 was provided in our previous study (22) where we showed that the distance between the fluorophores at positions –16 and +16 on the promoter DNA progressively decreased with each nucleotide addition to the RNA (Figure 9). Our studies here show that the C-tail region is essential for stabilizing the scrunched DNA throughout transcription initiation. Accordingly, stalled complexes of Rpo41 with Mtf1-WT showed a progressive increase in FRET levels from +2 to +7. However, such an increase in FRET levels was not observed with Mtf1-Δ12 in the present study. The FRET levels in +2 to +7 stalled complexes with Mtf1-Δ12 were low and broadly distributed. This indicated structurally distinct initiation complexes of Mtf1-Δ12, where the transcription bubble was less scrunched/bent and more flexible. These results provided evidence that the C-tail region of Mtf1 is critical for stabilizing the scrunched DNA during transcription initiation.

In addition to DNA scrunching, we find that the C-tail region is important for the timely transition into elongation. Typically, transition from initiation to elongation occurs after 8-mer synthesis (31,35), which assures that the RNA–DNA hybrid stable to support the RNA elongation process. This process in Mtf1-WT complexes started at position +8 with a sudden decrease in FRET level, followed by a gradual increase in the rates of upstream bubble collapse between positions +8 and +10 (22). In contrast, the FRET level in the Mtf1-Δ12 complexes at position +8 remained low and the upstream bubble region remained open and did not collapse until position +10. Thus, C-tail deletion in Mtf1 altered the trigger time for the transition into elongation. A comparison of the open and elongation complexes of POLRMT (13,36) shows that during transition into elongation the promoter DNA unbends and repositions to the same space as the C-terminal domain of TFB2M (Supplementary Figure S6). This indicates that the sudden decrease in FRET level at position +8 with Mtf1-WT is due to promoter unbending after the C-terminal domain releases the upstream promoter contacts.

Critical insight into the trigger mechanism for the transition into elongation was obtained from our observation that Mtf1 C-tail deletion mutants produce low amounts of abortive RNA products. These results are consistent with the presence of the Mtf1 C-tail in the path of the nascent RNA and suggest that steric clashes between the C-tail and RNA–DNA hybrid result in abortive synthesis when the RNA–DNA hybrid is short and energetically weak. However, as the RNA–DNA hybrid gets longer and more stable, the clashes reorganize the C-tail region, and after 8-mer synthesis, the C-tail region may eventually dissociate from the active site weakening upstream promoter contacts. Thus, C-tail region ejection may trigger the release of upstream promoter contacts and initiate the transition into elongation. The timing of upstream promoter release could be controlled by the relative strengths of the C-tail region interactions in the active site and the stability of the RNA–DNA hybrid. Such a mechanism assures that transition from initiation to elongation occurs only when the RNA–DNA hybrid reaches a stable length to support RNA elongation. A similar mechanism has been proposed for the bacterial RNAP with the σ3.2 region clashing with the RNA–DNA hybrid (40).

Lacking steric clashes in Mtf1-Δ12 complexes, the upstream promoter contacts remain stable until the RNA–DNA hybrid reaches 10-bp in length when we observe upstream bubble collapse in this mutant. Perhaps by the time the RNA–DNA hybrid is 10-bp long, the 5′-end of the RNA transcript naturally begins to thread into the RNA channel, which triggers upstream promoter release in the Mtf1-Δ12 complexes. Unassisted by the trigger from the full-length C-tail region, the transition process is inefficient and leads to a higher accumulation of 8–12 mer RNAs in the transcription reactions with the Mtf1 C-tail deletion mutants (Figure 2C, D and Supplementary Figure S3).

Overall, our findings provide detailed insights into the mechanism of transcription by the human and yeast mitochondrial RNAPs while revealing new and specific roles of the flexible C-tail regions of the initiation factors. The similarity in the functions of the C-tail and σ3.2 finger or B-reader of multisubunit RNAP factors is remarkable. These elements are evolutionarily unrelated, but in the initiation complex, they are located in a similar position near the active site between the melted DNA strands and performing the same functions during initiation (18,19). The σ3.2 finger regulates initiating nucleotide binding, RNA priming, and abortive products (20,21) as well as pausing during transcription initiation (41). This is an example of mechanistic convergence where RNAPs across different kingdoms have evolved a flexible element in their initiation factors that can be placed in the active site to anchor the template DNA for promoter-specific RNA synthesis and easily displaced when needed to bring about a timely transition into the elongation stage.

## Supplementary Material

gkaa040_Supplemental_File
