# Supplementary material for: The C-terminal tail of the yeast mitochondrial transcription factor Mtf1 coordinates template strand alignment, DNA scrunching and timely transition into elongation
Source: Nucleic Acids Res. 2020 Jan 25;48(5):2604–20. doi: 10.1093/nar/gkaa040 (PMC7049685; doi:10.1093/nar/gkaa040)
Supplement: gkaa040_Supplemental_File [file gkaa040_supplemental_file.pdf]

The C-terminal tail of the mitochondrial transcription factor coordinates template strand alignment, DNA scrunching, and timely transition into elongation

Supplementary Figures

Figure S1. Detailed view of the TFB2M C-tail interactions with POLRMT in the open complex. The C-tail of TFB2M (in red) is shown with amino acid side chains in stick mode that interact with the β-hairpin and thumb domain of POLRMT in the open complex (PDB:6ERP) with the locations of the C-tail deletion mutants. The Thr392 of the C-tail is within 3 Å distance from Arg770 in the thumb domain. There are 4 amino acids missing from the C-terminus of TFB2M in this structure.

Figure S2: Effect of Mtf1 C-tail deletion on the equilibrium binding of Rpo41-Mtf1 and promoter DNA. (A) The 4-20 % acrylamide SDS-PAGE of purified Mtf1-WT and deletion mutants with a molecular size ladder for reference. (B) Cartoon showing the basic scheme of the fluorescence anisotropy assays to monitor protein-DNA binding. (C) TAMRA-labeled -12 to +8 15S AA promoter (5 nM) was titrated with increasing Rpo41+Mtf1 (1:1.2 ratio). Fluorescence anisotropy was measured after excitation at 555 nm and emission at 580 nm. Titration data were fit to the quadratic equation 1 (Methods) to derive the Kd values listed in the table.

Figure S3. Transcription reactions on *15S* rRNA AA promoter with Mtf1-WT and C-tail deletion mutants. (A) Sequence of the *15S* rRNA AA promoter. (B) The 24% polyacrylamide-urea sequencing gel image shows the transcription profile for Mtf1-WT and C-tail deletion mutants with 2 to 8/12-mer abortive products and the 31-mer run-off RNA. Reactions were carried out with 1 µM Rpo41, 2 µM Mtf1-WT or deletion mutants, and 2 µM promoter DNA with 500 µM ATP (+γ[^{32}P] ATP), UTP, GTP and 3'dCTP at 25 °C. Lane 1, γ[^{32}P] ATP showing the impurities in that ATP batch. Lane 22, pre-quenched control sample, where 400 mM EDTA quencher was added to the reaction prior to the addition of NTPs. (C) and (D) RNA products (moles per mole of enzyme-

DNA complex) as a function of time were fit to calculate the rates of RNA synthesis. Standard errors from the linear fitting of data are shown. (E) The rate of longer 8-12 mer abortive synthesis is plotted for Mtf1-WT and C-tail deletion mutants (F) The ratio of 2-12 mer abortives to productive 31-mer for WT Mtf1 and mutants (D refers to delta). Error bars show the standard deviation from different time intervals.

Figure S4. Comparison of 2-mer synthesis efficiency of Mtf1-WT, Mtf1-Δ12, and Mtf1-Δ20. 2-mer synthesis was measured on the *21S* rRNA AG promoter at increasing concentration of equimolar ATP+GTP. Transcription reactions were carried out at 25°C using 1 µM Rpo41, 2 µM Mtf1-WT or deletion mutant, and 2 µM of promoter DNA in reaction buffer A. Reactions were stopped after 15 min with 98% formamide, 0.025% bromophenol blue, 10 mM EDTA. Samples were heated to 95°C for 2 min, chilled on ice, and the RNA products were resolved on 24% sequencing gel containing 4 M urea.

Figure S5. Efficiency of A-ladder synthesis by TFB2M-WT and TFB2M C-tail mutants on the LSP promoter. Transcription reactions were carried out with 1 µM each of POLRMT, TFB2M, TFAM, and LSP DNA at 25°C with increasing concentrations of ATP spiked with γ[^{32}P]ATP. The LSP promoter contains 3 A's at the start-site (shown bold underlined) and hence makes A-ladder in the presence of ATP alone. The A-ladders were resolved on a 40% acrylamide sequencing gel and grouped to calculate the rate. The initial slope of rate of A-ladder *versus* ATP concentration was used to calculate the catalytic efficiency or k_{cat}/K_m of RNA priming reaction. The error bars are derived from the standard error of fitting.

Figure S6. Promoter DNA in the open complex aligned with the DNA in the elongation complex. Open and elongation complexes (9-bp RNA-DNA) of POLRMT were aligned and two views of the aligned DNAs shown with TFB2M protein in blue. The green and blue strands of the DNA are the template and non-template strands of the LSP promoter DNA in the open complex of POLRMT (PDB: 6ERP), and the DNA strands in orange and RNA in magenta are from the elongation complex of POLRMT (PDB: 4BOC). The aligned structural view in (A) shows that the

promoter DNA is highly bent in the open complex and unbends in the elongation complex. The aligned view in (B) shows that the upstream DNA unbends upon transition to the elongation complex and occupies the same position as the C-terminal domain of TFB2M.

Figure S7. Mtf1-Δ12 is not capable of progressing to elongation at position +8. (A) DNA template design used for distinguishing elongation complex from open promoter state. Dye labeling positions are highlighted in magenta (Cy5) and green (Cy3). Promoter sequence is underscored and transcription start site is marked with an arrow. The complex can be stalled at positions +7 and +8 using combinations of nucleotide substrates as shown in the schematic. (B) FRET histograms of DNA only, open promoter complex, initiation complex at position +7, and elongation complex at position +8, compared between Mtf1-WT (blue) and Mtf1-Δ12 (brown). Dashed lines indicate the conformational states identified from our analysis. Note the overlay of blue and brown colors makes the dark shaded region in the histograms.

Figure S8. An alternative template sequence recapitulates that Mtf1 C-tail stabilizes the scrunched initiation complex. (A) Schematic design of an alternative DNA template II-2 to stall transcription at positions +7 and +8. Two AT base-pairs (shown in red) were swapped from DNA template II (red). (B) FRET histograms at each stalling position measured with Mtf1-WT (blue) and Mtf1-Δ12 (brown).

Figure S9. Kinetics of initial DNA bubble collapse compared for Mtf1-WT and Mtf1-Δ12 complexes. The cartoon shows the high fluorescence of the 2AP at position –4 in the +7 initiation complex and the expected low fluorescence when –4 position 2AP reanneals with the complementary base upon transitioning to the +8 elongation complex. DNA templates III and IV modified with 2AP at position –4 were used in the initiation bubble collapse experiments. The experiment was performed with 200 nM DNA and 400 nM each of Rpo41 and Mtf1-WT or Mtf1-Δ12. Transcription reactions were stalled at positions between +7 and +10 by adding combinations of NTPs and 3'dNTP, and 2AP fluorescence was measured for 800 s.

Figure S1

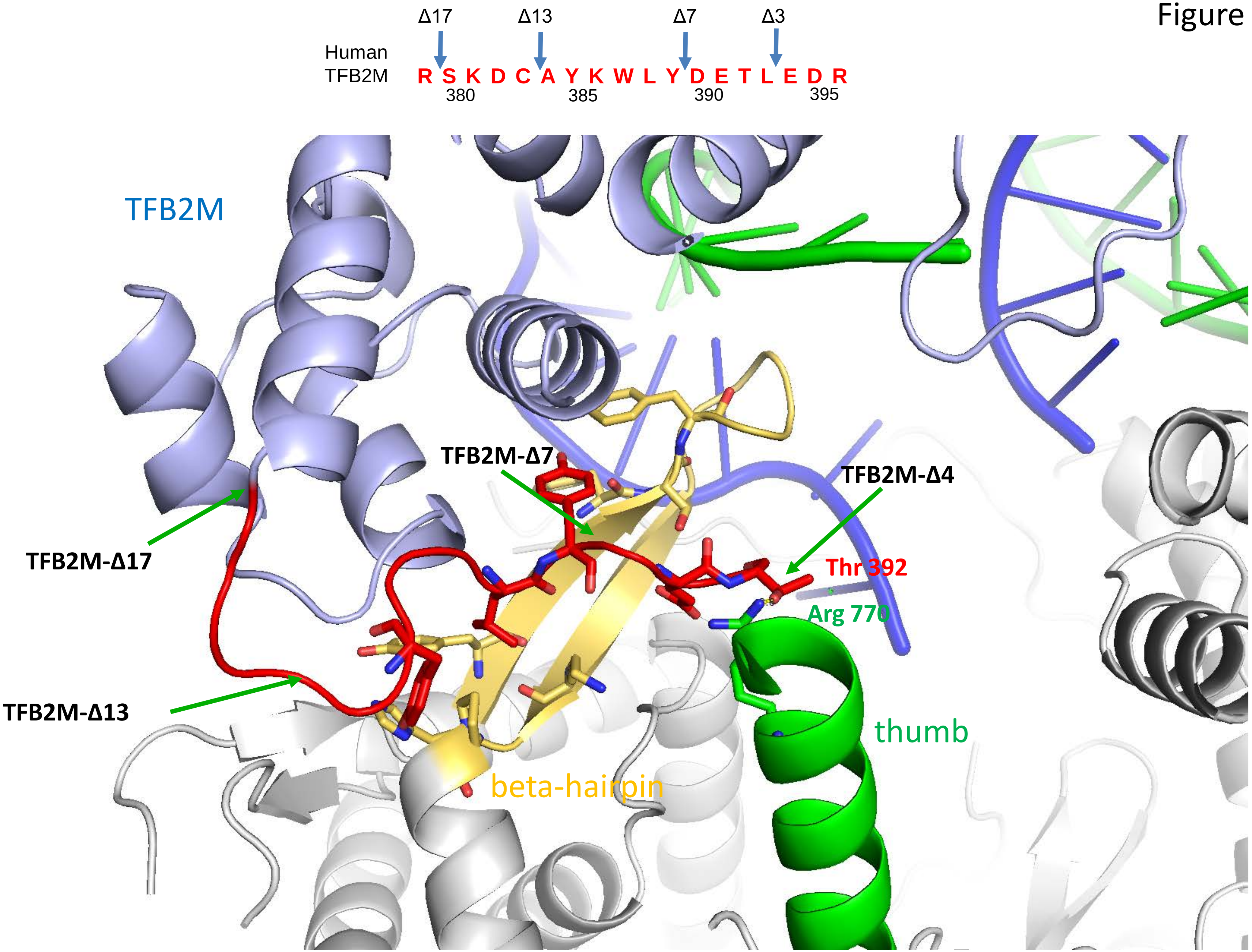
Δ17
Δ13
Δ7
Δ3
Human TFB2M
R S K D C A Y K W L Y D E T L E D R
380
385
390
395
TFB2M
TFB2M-Δ7
TFB2M-Δ4
TFB2M-Δ17
TFB2M-Δ13
Thr 392
Arg 770
thumb
beta-hairpin

Figure S2

A

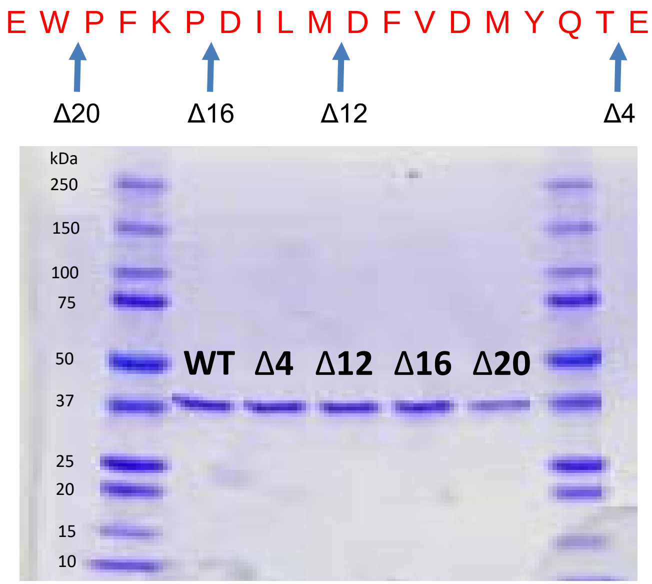

B

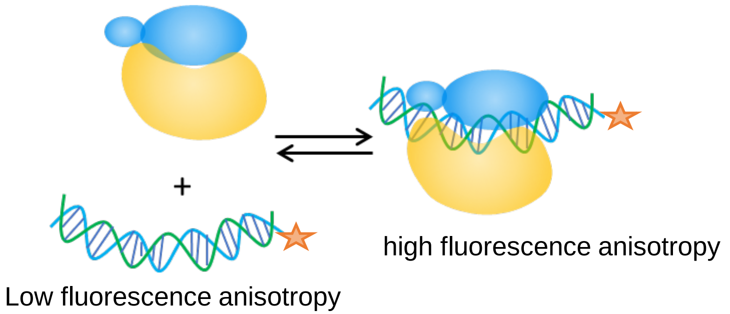

C

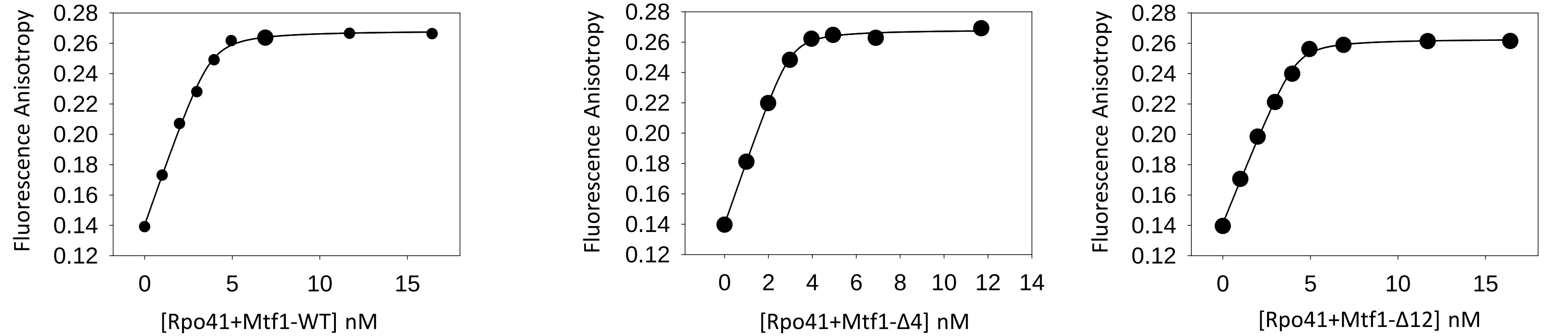

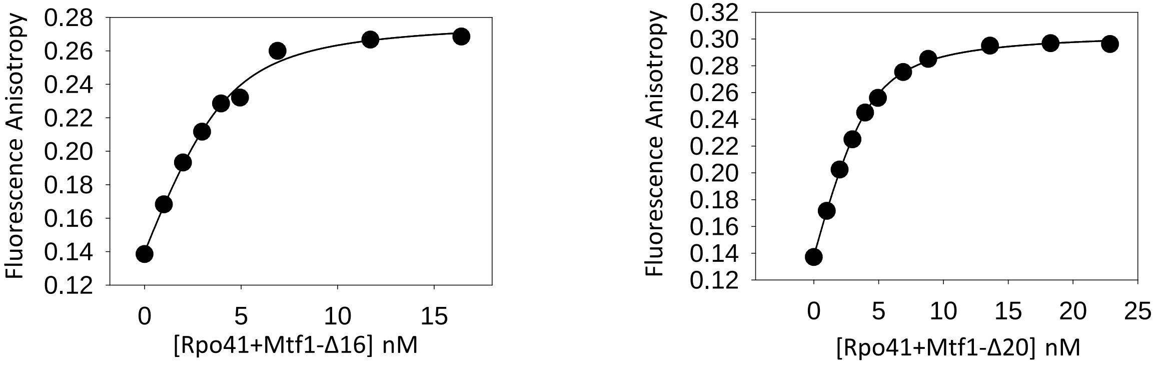

Mtf1	K_d (nM)
WT	0.12(±0.05)
Δ4	0.07(±0.03)
Δ12	0.09(±0.04)
Δ16	0.83(±0.50)
Δ20	0.81(±0.1)

Figure S3

A

```
                         -25                 -8       +1                              +32
15s NT: 5'ATAATTTATTTATTATTATATAAGTAATAAATAATTGTTTTATATAATAAGAATTCC
15s T:  3'TATTAAATAAATAATAATATATTCATTATTTATTAACAAAATATATTATTCTTAAGG
```

B

	1	2	3	4	5	6	7	8	9	10	11	12	13	14	15	16	17	18	19	20	21	22
Mtf1		WT				Δ4				Δ12				Δ16				Δ20				
t(min)	-	10	20	30	40	10	20	30	40	10	20	30	40	10	20	30	40	10	20	30	40	-

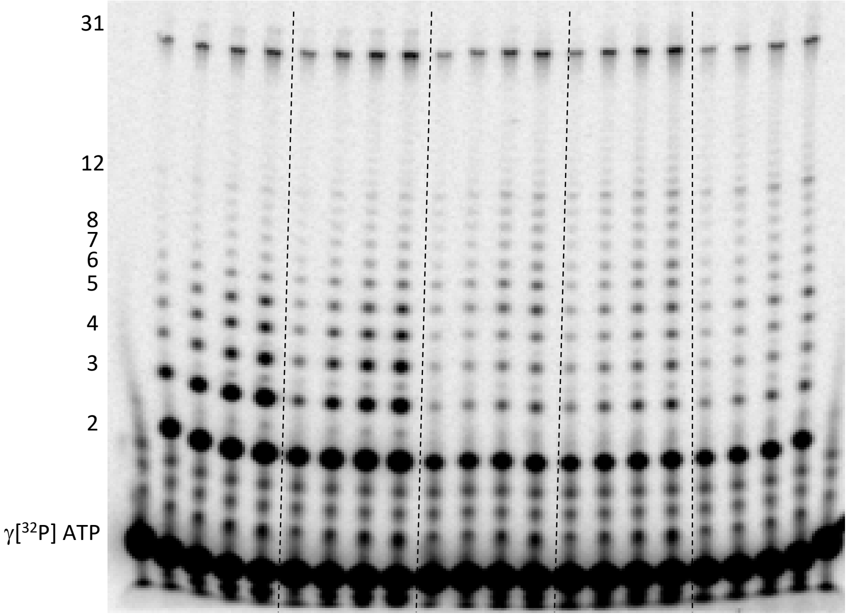

C

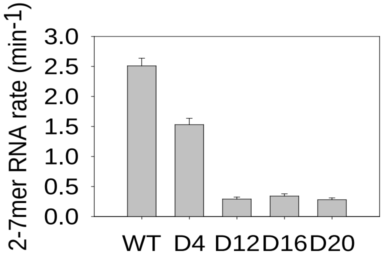

D

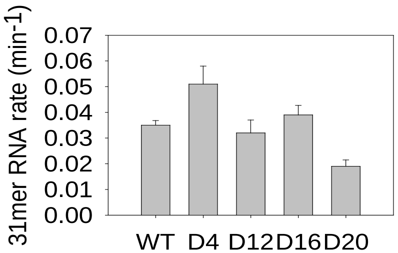

E

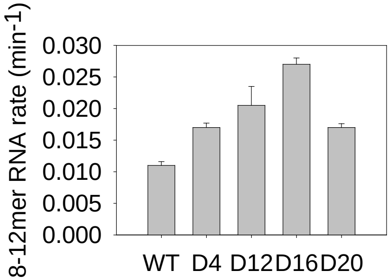

F

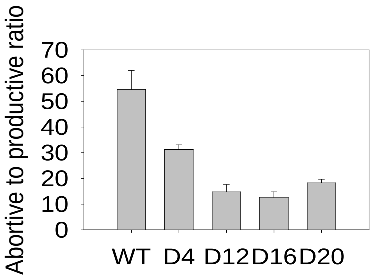

Figure S4

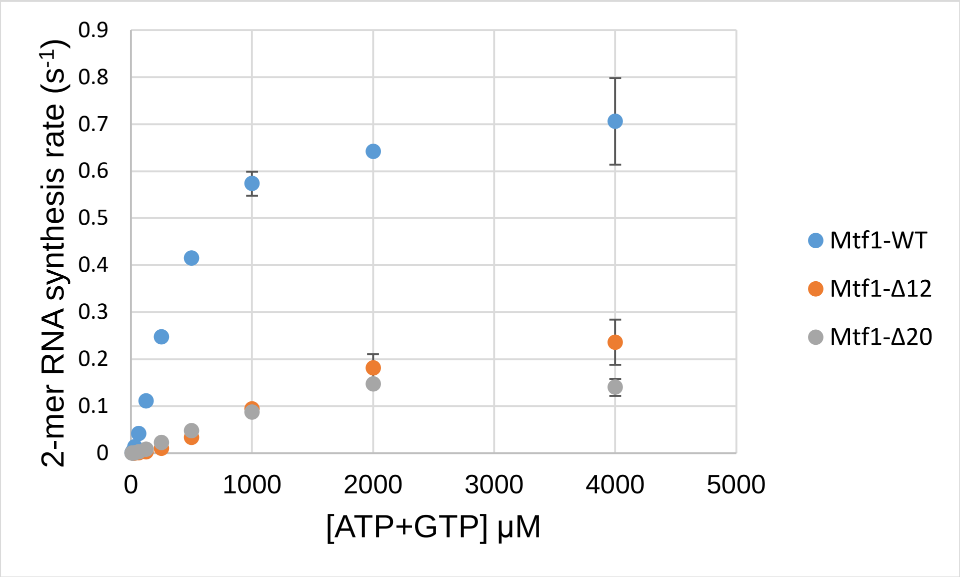

0.9
0.8
0.7
0.6
0.5
0.4
0.3
0.2
0.1
0
2-mer RNA synthesis rate (s-1)
0
1000
2000
3000
4000
5000
[ATP+GTP] μM
Mtf1-WT
Mtf1-Δ12
Mtf1-Δ20

Figure S5

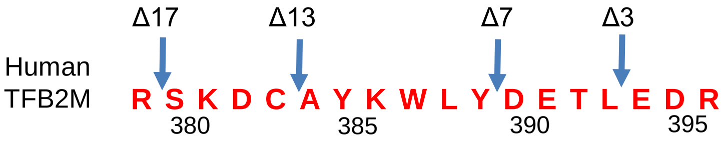

LSP (-42 TO +21)

```
NT 5'ATGTGTTAGTTGGGGGGTGACTGTTAAAAGTGCATACCGCCAAAAGATAAAATTTGAAATCTG
T  3'TACACAATCAACCCCCCACTGACAATTTTCACGTATGGCGGTTTTCTATTTTAAACTTTAGAC
```

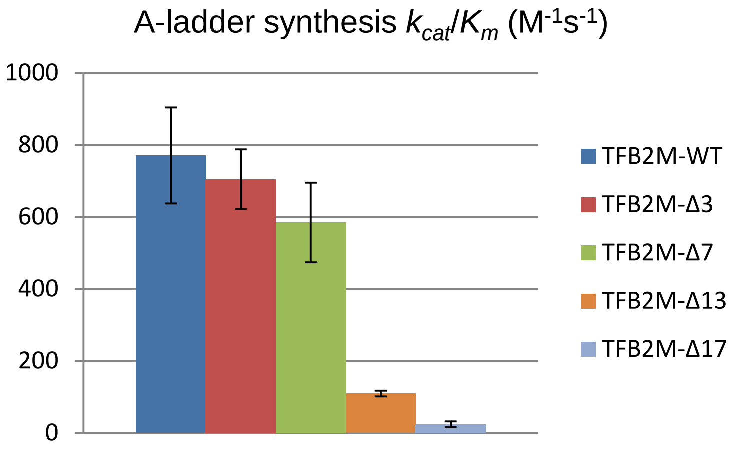

Figure S6

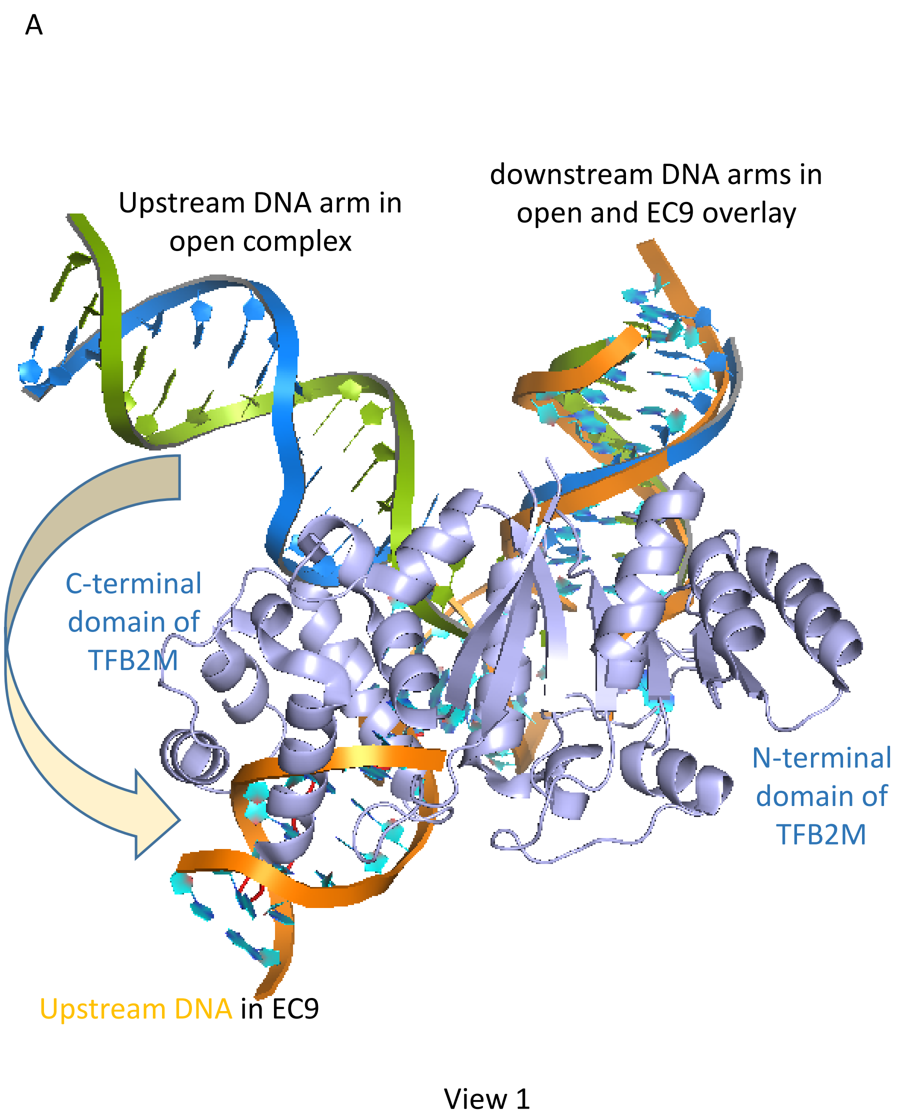

A
Upstream DNA arm in open complex
downstream DNA arms in open and EC9 overlay
C-terminal domain of TFB2M
N-terminal domain of TFB2M
Upstream DNA in EC9
View 1

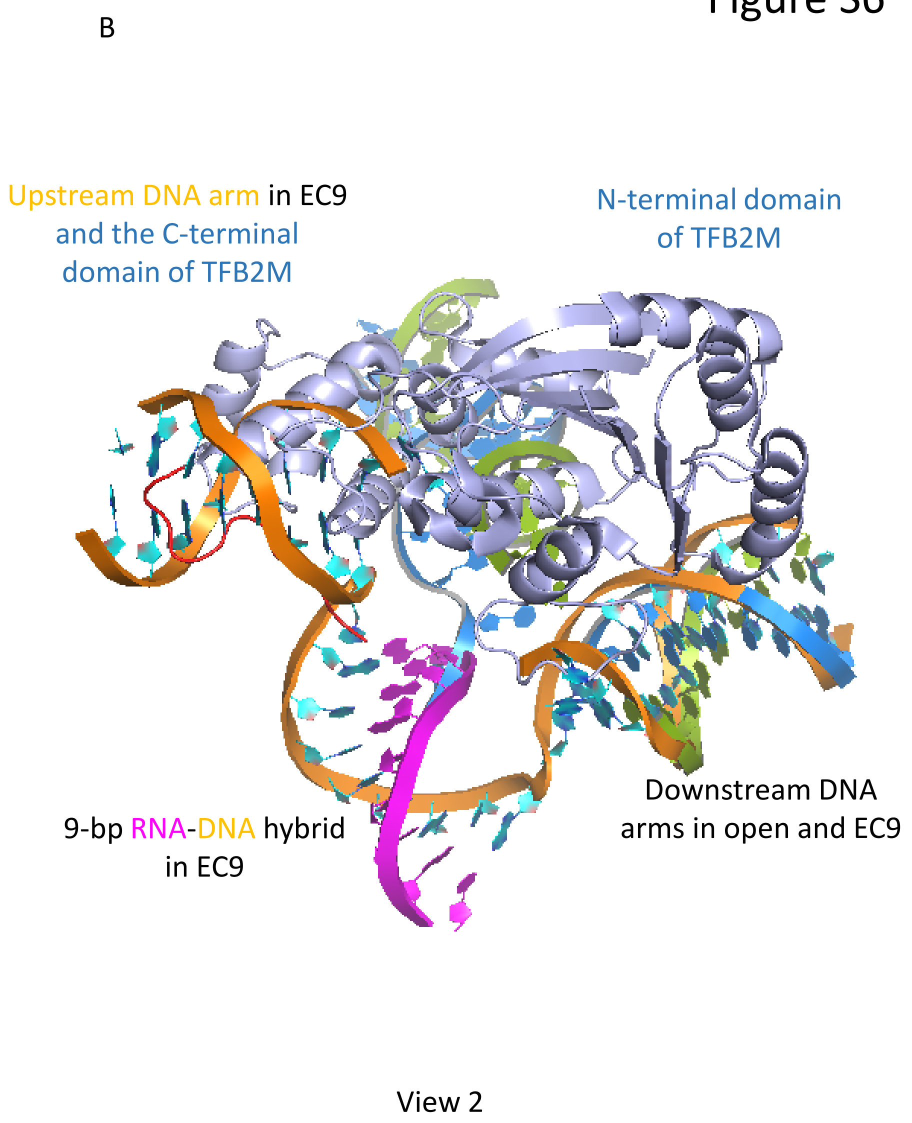

B
Upstream DNA arm in EC9 and the C-terminal domain of TFB2M
N-terminal domain of TFB2M
9-bp RNA-DNA hybrid in EC9
Downstream DNA arms in open and EC9
View 2

Figure S7

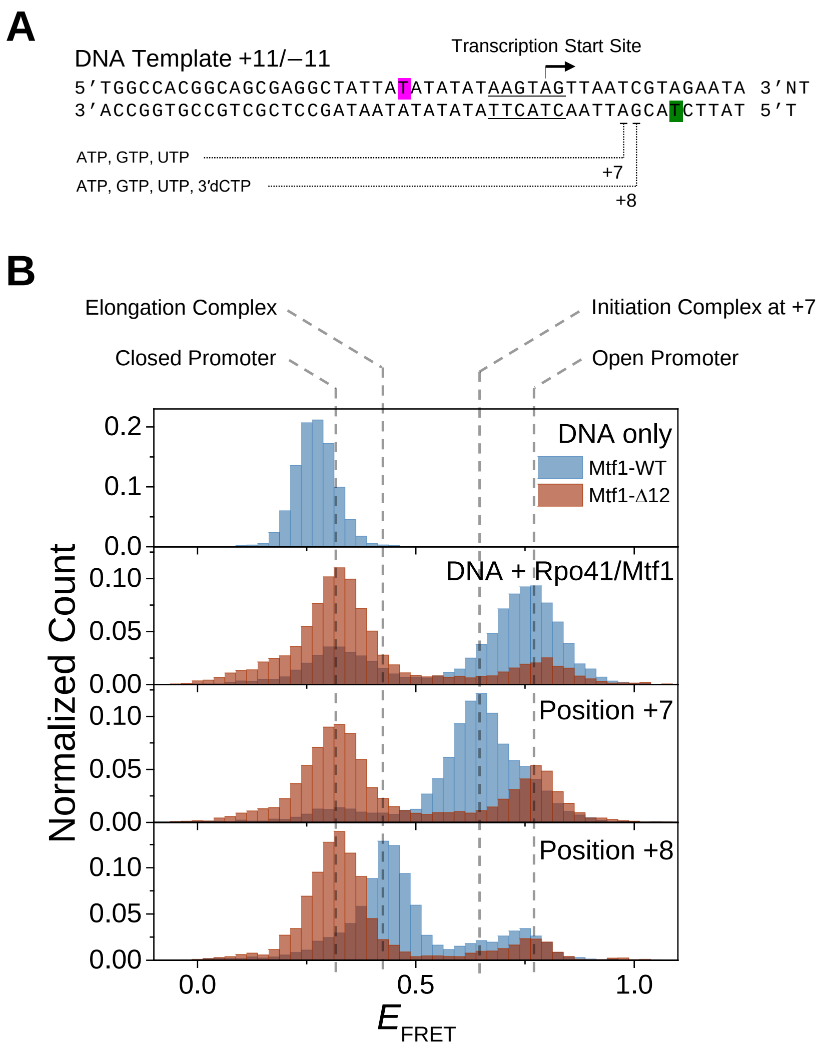
A
DNA Template +11/−11
Transcription Start Site
5′TGGCCACGGCAGCGAGGCTATTATATATATAAGTAGTTAATCGTAGAATA 3′NT
3′ACCGGTGCCGTCGCTCCGATAATATATATATTCATCAATTAGCATCTTAT 5′T
ATP, GTP, UTP
+7
ATP, GTP, UTP, 3′dCTP
+8
B
Elongation Complex
Initiation Complex at +7
Closed Promoter
Open Promoter
DNA only
Mtf1-WT
Mtf1-Δ12
DNA + Rpo41/Mtf1
Position +7
Position +8
Normalized Count
E FRET
0.0
0.5
1.0

Figure S8

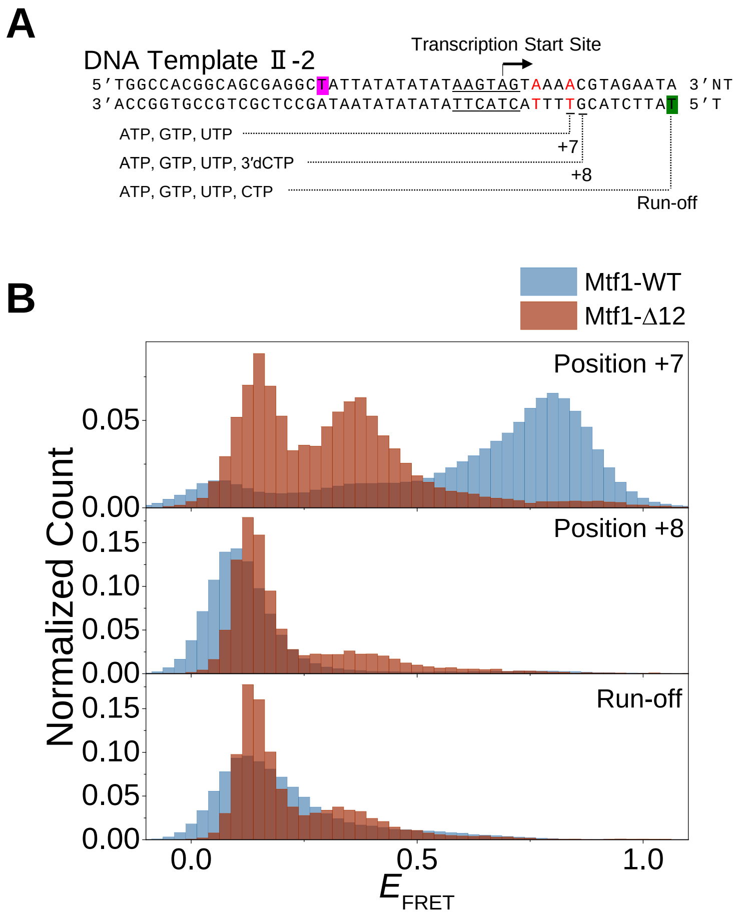
A
DNA Template Ⅱ-2
Transcription Start Site
5′TGGCCACGGCAGCGAGGCTATTATATATATAAGTAGTAAAACGTAGAATA 3′NT
3′ACCGGTGCCGTCGCTCCGATAATATATATATTCATCATTTTGCATCTTAT 5′T
ATP, GTP, UTP
+7
ATP, GTP, UTP, 3′dCTP
+8
ATP, GTP, UTP, CTP
Run-off
B
Mtf1-WT
Mtf1-Δ12
Position +7
Position +8
Run-off
Normalized Count
0.00
0.05
0.10
0.15
0.0
0.5
1.0
E FRET

Figure S9

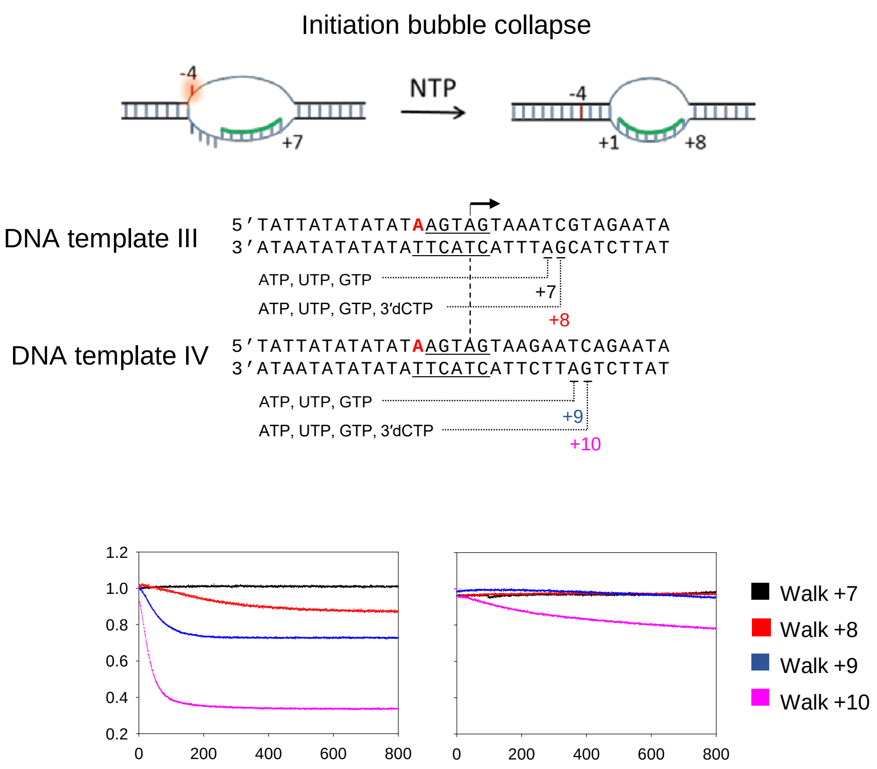

Initiation bubble collapse
-4
+7
NTP
-4
+1
+8
DNA template III
5′TATTATATATATAAGTAGTAAATCGTAGAATA
3′ATAATATATATATTCATCATTTAGCATCTTAT
ATP, UTP, GTP
+7
ATP, UTP, GTP, 3′dCTP
+8
DNA template IV
5′TATTATATATATAAGTAGTAAGAATCAGAATA
3′ATAATATATATATTCATCATTCTTAGTCTTAT
ATP, UTP, GTP
+9
ATP, UTP, GTP, 3′dCTP
+10
1.2
1.0
0.8
0.6
0.4
0.2
0
200
400
600
800
Walk +7
Walk +8
Walk +9
Walk +10

Table S1: Kinetic parameters for 2-mer synthesis on AG and AA promoters

**+1+2 for AG**	**Mtf1-WT**	**Mtf1-Δ20**
K_m (μM)	580 (±90)	1450 (±240)
k_{cat} (s^{-1})	0.84 (±0.06)	0.2 (±0.02)
k_{cat}/K_m ($M^{-1}s^{-1}$)	1460 (±126)	150 (±10)
**+1+2 for AA**	**Mtf1-WT**	**Mtf1-Δ20**
K_m(μM)	165 (±20)	364 (±64)
k_{cat} (s^{-1})	0.4 (±0.1)	0.25 (±0.01)
k_{cat}/K_m ($M^{-1}s^{-1}$)	2412 (±674)	686 (±124)

Table S2. FRET transition rates (s^{-1}) for Mtf1-WT and Mtf-Δ12 obtained from hidden Markov analysis.

	**Opening Rate**	**Closing Rate**	**Scrunching Rate**	**Unscrunching Rate**
Mtf1-WT	0.095 ± 0.003	0.73 ± 0.02	0.18 ± 0.02	0.30 ± 0.004
Mtf1-Δ12	0.42 ± 0.007	0.63 ± 0.01	0.73 ± 0.01	0.47 ± 0.008
